# Bacterial metagenome profiling of hand-made herby cheese samples utilizing high-throughput sequencing to detect geographical indication and marketing potential

**DOI:** 10.1016/j.heliyon.2023.e13334

**Published:** 2023-02-09

**Authors:** Mustafa Rüstemoğlu, Mehmet Emin Erkan, Gazal Cengiz, Mortaza Hajyzadeh

**Affiliations:** a Şırnak University, Faculty of Agriculture, Plant Protection Department 73300 İdil, Şırnak, Turkey; b Dicle University, Faculty of Veterinary Medicine, Department of Food Hygiene and Technology, 21280 Diyarbakir, Turkey; c Şırnak University, Faculty of Health Sciences, Department of Health Management, Şırnak, Turkey; d Şırnak University, Faculty of Agriculture, Field Crops Department 73300 İdil, Şırnak, Turkey

**Keywords:** Herby cheese, Turkey, *Companilactobacillus ginsenosidimutans*, Microbiota, Geographical indications, NGS

## Abstract

Food safety has been a major concern for consumers. Origin of food products matter for consumers such that the quality, reputation, or other special characteristics can be attributed essentially to that origin. While a geographical indication informs consumers for the origin of the product, it develops a competitive advantage for the markets. To detect distinguishing features of dairy products, the microbial composition of its microbiota is one of the emerging areas of interest. Utilizing novel approaches such as Next Generation Sequencing (NGS) technology to decipher the genetic code of 16s rRNA genes to characterize the bacterial population is widely applied. The bacterial microbiota of the herby cheese samples which were collected from Şırnak province in the South Eastern region of Turkey was examined by an NGS approach for purpose of finding geographical indication possibilities. In brief, Firmicutes is the dominant phyla where Lactobacillaceae and Streptococcaceae are abundant families across the analyzed herby cheese microbiota. The most prominent species is *Companilactobacillus ginsenosidimutans* detected as the dominant member of the bacterial consortia in 16 herby cheese samples. Another remarkable finding reported here is the *Weissella jogaejeotgali* which was detected in 15 cheese samples. Albeit the abundance of *Levilactobacillus koreensis* is low at the microbiome level it was identified in four herby cheese samples. As expected, lactic acid bacteria such as *Lactobacillus delbrueckii*, *Lactococcus raffinolactis* and *Tetragenococcus halophilus* were also identified. On the other hand, bacterial diversity and microbial composition among cheese samples are not significantly affected by mixing different herbs on the manufacturing of herby cheeses. To the best of our knowledge, *C. ginsenosidimutans*, *W. jogaejeotgali* and *L. koreensis* are identified and reported for the first time in a dairy product and the bacterial richness and evenness of herby cheese are higher than those of most other cheeses. These findings make the cheeses in the geography where the samples were produced more valuable and provide opportunities for them to receive geographical indications. Thus, it will create added value while marketing the products.

## 1 Introduction

Consumer perception and attitudes has been major subjects for marketing discipline. Consumer behavior and needs changed with globalization. Markets got closer, foreign products entered local markets and information is accessed by consumers faster. These advances triggered a change in consumer perceptions and lifestyles, and they moved away from traditional consumption [1]. On the other hand, industrial food production created food safety as a major matter worldwide. Geographical indication (GI) is considered one of the best ways to gain consumer trust and competitive advantages for producers. Competitiveness of local products is considered for the development of a certain region and important for economic activity to compete with foreign markets and importing goods. GI helps the market recognition, premium capture, creates exoticness or scarcity images, differentiate their products from others and gain legal protection. GI also provides local agricultural development as it is recognized as a qualification strategy for the specific socio-cultural territory [2]. Food safety and inspected label effect positively the willingness of consumers for paying more [3] because they see industrial food harmful for health and environment [4].

Origin of products matters for consumers [5]. GI is a sign used on products that have a specific geographical origin and possess qualities or a reputation that are due to that origin. To function as a GI, a sign must identify a product as originating in a given place [6]. GI became more popular in marketing of food products. To detect the distinguishing features for food products it is essential to get assistance of biological traces. In this study, microbiota of cheese researched to reveal GI potential to be used for marketing purposes. Market access and differentiation emerge as two main factors for success and origin-labeled variolization strategy develop a competitive advantage by working with these two factors [7].

Herby cheese is one of the popular types of cheese frequently consumed, in the south-eastern regions of Turkey. It is semi-hard and salty, usually produced by the local people living in the region for in-house consumption or marketing at a small-scale level. Although raw sheep milk is mostly used in the production of traditional herby cheese, it can also be mixed with cow or goat milk. Manufacturing of herby cheese is started in May and June then its ripening takes place underground for a while [8,9]. A single herb or even more than twenty kinds of herbs are used in herby cheese manufacturing. The herb species differs according to the geographical region where the cheese is produced. Some of the herbs used in the herby cheese are *Allium* sp., *Chaerophyllum macrospermum* [10], *Diplotaenia turcica* [11], *Heracleum persicum* [12]. Depending on the species used in the production, leaf, stem, or both of the herbs are added to the herby cheese.

To date, several studies were carried out on the characteristic properties of herby cheese. Studies based on chemical content analysis such as dry matter, fat, protein and pH were conducted [[13], [14], [15], [16]]. Mineral content of herby cheese samples were reported elsewhere [17,18]. In addition to these studies, the total bacterial load of herby cheese including total aerobic, mesophilic, lactic acid, coliform bacteria as well as yeast and mold contents were counted [19].

Recent advances in the usage of Next Generation Sequencing (NGS) technologies deeply affected food microbiology. Cost-effective and rapid production of genomic information let by NGS provided an opportunity to understand the bacterial composition of foods in detail [20]. With this advance, several studies were performed on 16 s rRNA gene metagenome analysis for dairy products including kefir [21], yogurt [22], Siciliano cheese [23], Feta cheese [24], Cheddar, provolone and Swiss cheeses [25], and Kazakh cheese [26]. On the other hand, there is only one study conducted to examine the microbial diversity of herby cheese using the NGS approach [27].

Herein, a detailed metagenome study on the herby cheese samples was aimed. In order to determine the effect of location and herb species on bacterial diversity and richness 33 herby and three non-herby cheese samples were collected from seven sampling points in Şırnak province of Turkey.

## 2 Material and methods

### 2.1 Cheese samples

Homemade produced herby cheese samples subjected to metagenome analysis were collected from the provincial borders of Şırnak, Turkey (Table 1). The local manufacturing process of the herby cheese as follows; unpasteurized sheep milk was immediately fermented for 1 h using a starter culture. The specified plant leaves given in Table 1 were mixed with cheese samples. Then, the mixture was placed in cheesecloth and a weight was placed on it to drain the juice off for a day. On the next day, cheese was sliced and moved to saltwater for a week to solidify. Later on, the cheese was placed firmly in a plastic jar closing the lid tightly. For ripening, the cheese in the jar was buried into the soil for up to six months. Cheese samples were collected from 36 different localities of Şırnak province (Table 1). Here, we also analyzed non-herby cheese samples such as Sample 1 which was ripened in sheepskin, Sample 3, a curd cheese, and other two cheese samples which were manufactured as herby cheese without mixing any plant, Sample 5 and Sample 16 (Table 1). The collected cheese samples were transported to the sequencing laboratory in a foam box filled with dry ice in sterile tubes. Samples were stored in a freezer at −20 °C until analysis.

**Table 1:** Localization of the cheese samples and herbs used to make herby cheese.

Cheese Samples	Addition of herbs (Yes/No)	Localization	Herbs involved in cheese production	
			Scientific name	Local name of herbs
1	No	İdil	Cheese ripened in sheepskin	–
2	Yes		*Allium vineale* L.	Sirik
3	No		Curd cheese	–
4	Yes		*Heracleum persicum* Desf.	So
5	No		Herb-free cheese	–
6	Yes	Beytüşşebab	*Allium vineale* L.	Sirik
7	Yes		*Diplotaenia turcica* Pimenov & Kljuykov	Siyabu
8	Yes		*Allium vineale* L.	Sirik
9	Yes		*Diplotaenia turcica Pimenov & Kljuykov, Chaerophyllum macrospermum (Sprengel) Fisch. & C.A.Mey.*	Siyabu-Mende
10	Yes		*Diplotaenia turcica* Pimenov & Kljuykov	Siyabu
11	Yes		*Ferulago stellata* Boiss	Bük
12	Yes	Uludere-1	*Ferulago stellata* Boiss	Bük
13	Yes		*Allium vineale* L., *Allium cepa* L.	Sirik-Pivaz
14	Yes		*Ferulago stellata* Boiss	Bük
15	Yes		*Allium vineale* L.	Sirik
16	No		Herb-free cheese	–
17	Yes	Uludere-2	*Ferulago stellata* Boiss	Bük
18	Yes		*Allium vineale* L.*, Gundelia tournefortii* L. *var. tournefortii L. Gundelia colemerikensis Fırat.*	Sirik- Gerenk
19	Yes		*Allium cepa* L., *Ferulago stellata* Boiss	Pivaz-Bük
20	Yes		*Allium cepa* L.	Pivaz
21	Yes		*Allium vineale* L.	Sirik
22	Yes		*Allium cepa* L.	Pivaz
23	Yes		*Allium cepa* L.	Pivaz
24	Yes		*Allium cepa* L., *Chaerophyllum macrospermum* (Sprengel) Fisch. & C.A.Mey.	Pivaz-Mende
25	Yes		*Ferulago stellata* Boiss	Bük
26	Yes		*Ferulago stellata* Boiss	Bük
27	Yes		*Allium cepa* L., *Chaerophyllum macrospermum* (Sprengel) Fisch. & C.A.Mey.	Pivaz-Mende
28	Yes	Şenoba	*Allium vineale* L., *Diplotaenia turcica* Pimenov & Kljuykov	Sirik-Siyabu
29	Yes		*Chaerophyllum macrospermum (Sprengel) Fisch. & C.A.Mey., Gundelia tournefortii* L. *var. tournefortii L. Gundelia colemerikensis Fırat.*	Mende-Gerenk
30	Yes		*Allium vineale* L.*, Gundelia tournefortii* L. *var. tournefortii L. Gundelia colemerikensis Fırat.*	Sirik- Gerenk
31	Yes	Beytüşşebab	*Allium vineale* L.	Sirik
32	Yes		*Diplotaenia turcica* Pimenov & Kljuykov	Siyabu
33	Yes	Cizre	*Allium vineale* L.	Sirik
34	Yes	İdil	*Allium scorodoprasum* L. subsp. *rotundum* (L.) Stearn	Kurat
35	Yes	Şırnak (Center)	*Allium vineale* L.	Sirik
36	Yes	Beytüşşebab	*Ferulago stellata* Boiss	Bük

### 2.2 Bacterial genomic DNA extraction

Total genomic DNA was extracted using the Zymo Research Quick-DNA Fecal/Soil Microbe Miniprep Kit (Irvine, USA), following the manufacturer's recommendations. Quantitation of DNA was detected with Qubit dsDNA BR Assay Kit (Invitrogen, CA) according to the manufacturer's instructions, and stored at −20 °C prior to PCR.

### 2.3 PCR conditions of 16S V3–V4 region

16s rRNA gene V3–V4 region was amplified with universal primers (Alpha DNA, Canada) 341F (5′-CCTACGGGNGGCWGCAG-3′) and 805R (5′-GACTACHVGGGTATCTAATCC-3′) by using SimpliAmp Thermal Cycler (Thermo Fisher Scientific, USA). The 25 μl PCR reaction mixture consisted of 30 ng genomic DNA, 10 mM Tris-HCl (pH 8.5), 50 mM KCl, 1.5 mM MgCl_2_, 0.25 mM each dNTP, 1.5 U Hot-Start Taq DNA Polymerase and 0.2 μM each primer. PCR conditions were as follows: An initial denaturation at 95 °C for 5 min and then 35 cycles with denaturation at 95 °C for 30 s, annealing at 53-48 °C for 30 s, and extension at 72 °C for 30 s. A final extension was performed at 72 °C for 3 min. Amplification results were determined on a 2% agarose gel.

### 2.4 Preparing library and sequencing

16S rRNA gene V3–V4 amplicon products were purified by Column-Pure Gel and PCR Clean-Up Kit (ABMGood, CA). The library was prepared for 16S rRNA V3–V4 amplicon products with Nextera XT DNA Library Prep Kit (Illumina, USA). Indexing was carried out with TG Nextera XT Index Kit v2 Set A (96 Indices, 384 Samples) (Illumina, USA). Sequencing was performed as paired-end (PE) and 2 × 150 bp read lengths with the Illumina MiSeq platform (Illumina, USA).

### 2.5 Bioinformatic analysis

Raw NGS reads (FASTQ) were quality checked by FASTQC (Andrews 2010) and trimmed by Trimmomatic v0.32 (Bolger et al., 2014). Demultiplexing and low-quality read filtering were performed via CLC Genomics Workbench (Qiagen, US) Clean reads were classified based on OTU (operational taxonomic unit) criteria using the Kraken2 metagenomics system (Wood and Salzberg 2014). OmicsBox software (BioBam Bioinformatics, ES) was used to generate principal coordinate analysis (PCoA) plots based on Bray-Curtis distances. Heatmaps were created with the PermutMatrix v1.9.3 package with the “Euclidean” distance.

### 2.6 Shannon and Simpson diversity index

Shannon and Simpson diversity indices were calculated at the species level and used as a measure of species richness and evenness to quantify the diversity of the bacteria species in cheese samples. The Shannon diversity index usually takes a value between 1.5 and 3.5 and rises with the evenness. Simpson diversity index (1-D) is a value between 0 and 1, 1 corresponds to complete evenness.

## 3 Results

A total number of 1,456.598 paired reads were generated with an average length of 108.9 ± 3.87 bases with a quality of Q30 > 90%. Therefore, we have obtained approximately 40.461 paired reads per sample ranging from 7.062 to 182.625 (Table 2). To show bacterial diversity and species richness we have calculated the Shannon index values of the samples with an average of 3.03 ± 0.65 (range 1.81–4.81), and that of Simpson index as 0.83 ± 0.08 (range 0.63–0.97). The samples with the lowest Shannon and Simpson indices were Sample 32 (1.812, 0.6355), Sample 23 (1.831, 0.6569), Sample 12 (2.001, 0.6756), and Sample 19 (2.13, 0.6814) while the samples with the highest values were Sample 5 (4.81, 0.9792), Sample 7 (4.104, 0.9409) and Sample 35 (3.965, 0.9347). According to localizations, the sample with the highest Shannon and Simpson index was the cheese sample collected from the Şırnak (Center). The average of Shannon and Simpson indexes of cheese samples manufactured in the Uludere-2 region was the lowest compared to other regions (Table 3).

**Table 2:** Sequencing statistics, Shannon and Simpson indices of species-level diversity.

Cheese Samples	Number of reads	Average reading length (base)	Shannon Index (H)/(H/LN (N))	Simpson Index (1-D)
1	7,062	107.2	2.913/0.5434	0.8042
2	61,008	103.9	2.744/0.4573	0.7697
3	47,138	113.4	3.409/0.5854	0.8931
4	34,211	110.3	2.736/0.4481	0.775
5	39,491	107	4.81/0.7774	0.9792
6	36,561	106.6	2.612/0.4446	0.7435
7	56,645	111.9	4.104/0.6291	0.9409
8	22,420	112.7	2.84/0.5111	0.8458
9	34,123	110.7	3.034/0.5214	0.8394
10	27,509	109.3	2.931/0.5176	0.8282
11	29,223	109.8	3.11/0.5674	0.8693
12	34,664	108	2.001/0.3402	0.6756
13	42,756	109.1	2.18/0.3608	0.7066
14	13,213	110.7	3.534/0.6338	0.9205
15	35,152	112.2	2.794/0.4635	0.7977
16	20,350	114.3	3.593/0.5946	0.8965
17	45,877	110	2.861/0.4913	0.8354
18	180,625	108.7	2.533/0.4088	0.7726
19	56,215	115.1	2.13/0.3483	0.6814
20	30,248	110.3	3.35/0.5369	0.8448
21	24,298	95	2.625/0.4548	0.7967
22	59,239	111.4	2.368/0.3971	0.7767
23	47,622	108.1	1.831/0.3018	0.6569
24	52,759	105.8	2.609/0.4354	0.8273
25	76,105	109.7	3.41/0.5326	0.8995
26	75,343	109	3.161/0.5046	0.8222
27	12,497	105.7	3.433/0.5789	0.9044
28	41,543	104.3	2.788/0.4618	0.8371
29	16,207	106	3.334/0.5711	0.8964
30	34,035	105	2.847/0.4768	0.8384
31	49,632	104.7	3.753/0.6047	0.9328
32	25,194	109.4	1.812/0.3066	0.6355
33	21,273	109.6	3.532/0.6527	0.9332
34	7,460	112.4	3.921/0.651	0.9122
35	8,239	117	3.965/0.7208	0.9347
36	50,661	106.1	3.605/0.589	0.9117

**Table 3:** The Average Shannon and Simpson indexes of cheese samples according to collected localization.

Localization of cheese samples	Cheese Samples	Shannon Index Mean ± SD	Simpson Index Mean ± SD
İdil	1-5, 34	3.422 ± 0.748	0.8555 ± 0.0778
Beytüşşebab	6-11, 31,32, 36	3.089 ± 0.639	0.8385 ± 0.0919
Uludere-1	12–16	2.820 ± 0.661	0.7993 ± 0.0980
Uludere-2	17–27	2.755 ± 0.513	0.8016 ± 0.0745
Şenoba	28–30	2.989 ± 0.244	0.8573 ± 0.0276
Cizre	33	3.532	0.9332
Şırnak (Center)	35	3.965	0.9347

**Fig. 1:**
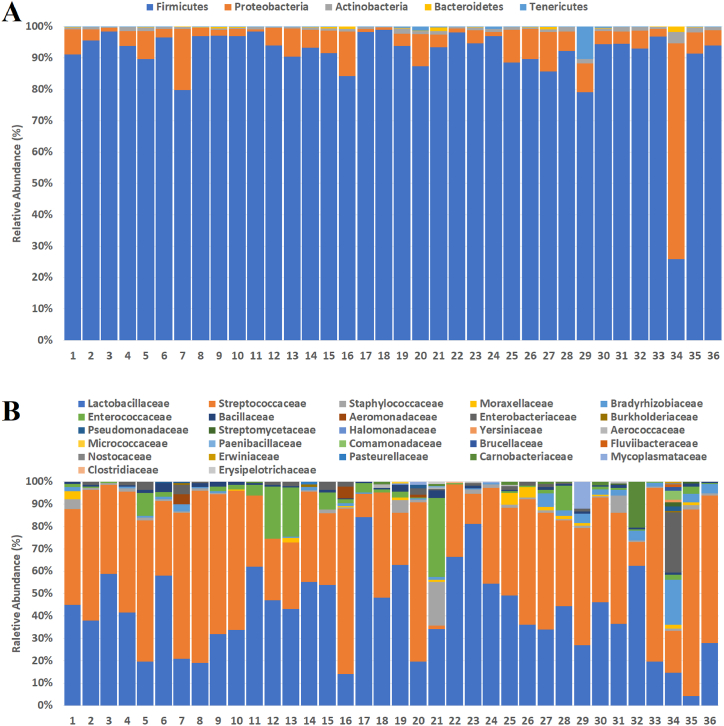
Distribution of bacterial communities. (A) Phylum level; (B) Family level.

Firmicutes were found to be the highly represented taxon at the phylum level in all samples except Sample 34 in which Proteobacteria is the dominant. Hence, Actinobacteria, Bacteroidetes, and Tenericutes are detected Firmicutes and Proteobacteria poses >97% of total relative abundance except two samples, Sample 29 and Sample 34 (Fig. 1A).

Each sample has a diversity of twenty-seven families at abundance >0.5%. Of which Lactobacillaceae and Streptococcaceae were the most prevalent two families with a co-prevalence range of 29–96%. On the other hand, abundance of Enterococcaceae family was detected in high level for such samples as Sample 21, Sample 12, Sample 13, Sample 28, Sample 5, and Sample 15 (33.68%, 22.64%, 21.33%, 10.55%, 9.81%, and 7.30%, respectively). Additional non-lactic acid bacteria families such as Enterobacteriaceae, Moraxellaceae, and Pseudomonadaceae, were observed in some cheese samples (Sample 13, Sample 25, and Sample 26) (Fig. 1B).

A total of 2.950 bacteria species were identified in all cheese samples. Of which 114 species were present with an abundance of at least 0.5% in at least one sample (Supplementary Table 1). According to the overall metagenomic analyses, *Companilactobacillus ginsenosidimutans* is the mostly encountered species which was highly abundant in 16 of 36 samples. The second highly represented species is *Lactobacillus delbrueckii*, which was dominant species in 7 of 36 samples. Following species are measured the most abundant species at least in one sample; *Streptococcus parauberis* (in six samples), *Bradyrhizobium* sp. PSBB068 (in three samples), *Streptococcus suis* (in two samples), *Tetragenococcus halophilu*s and *Lactococcus raffinolactis* (in one sample) (Supplementary Table 1).

On the other hand, following bacteria species were listed as second mostly represented in at least one sample; *Weissella jogaejeotgali, Streptococcus parauberis, Lactiplantibacillus plantarum, Staphylococcus aureus, Bacillus thuringiensis, Tetragenococcus halophilus, Lactococcus raffinolactis, Ligilactobacillus acidipiscis, Lactococcus cremoris, Bradyrhizobium* sp. PSBB068, *Companilactobacillus alimentarius, Marinilactibacillus* sp. 15R, *Lactococcus cremoris* (Supplementary Table 1).

**Fig. 2:**
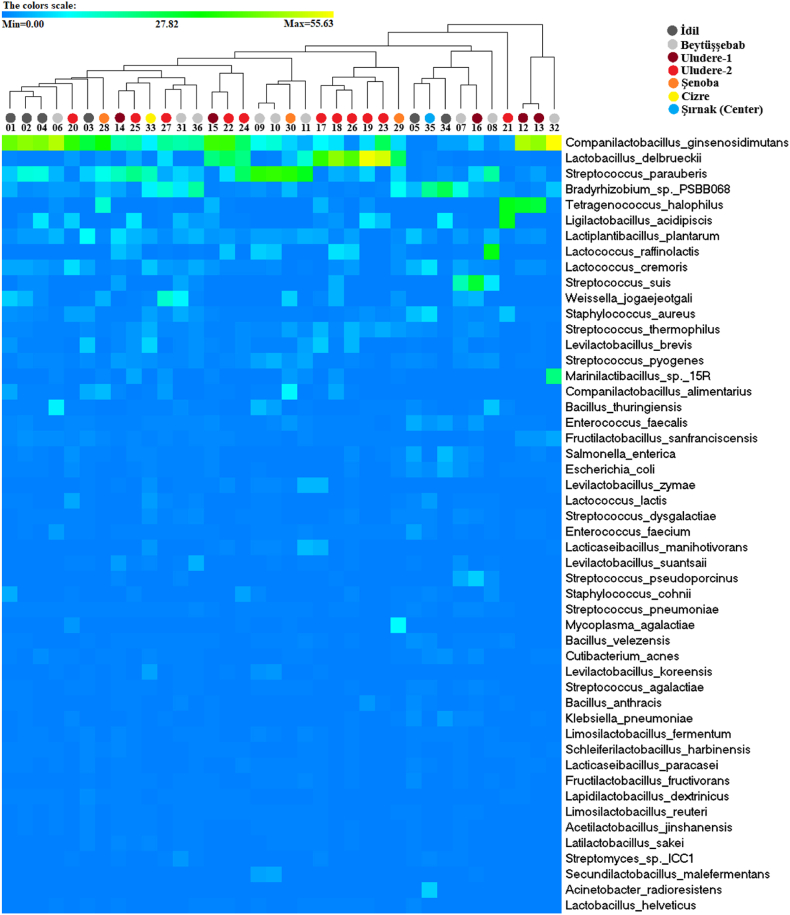
Community Heatmap based on the abundance of the 50 most represented species in the cheese samples. The columns correspond to the samples. Each row corresponds to the microbiota in species level. The hierarchical clustering was performed using the Euclidean distance.

**Fig. 3:**
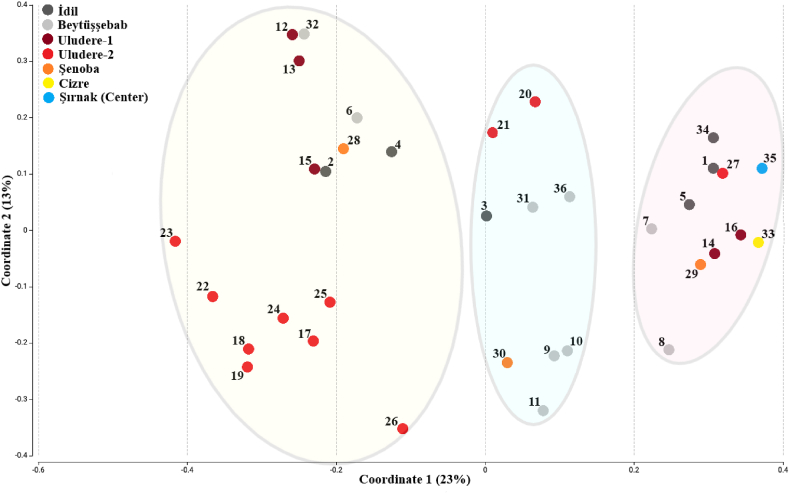
Principal coordinate analysis (PCoA) based on the overall structure of the microbiota in all cheese samples. Each data point represents an individual sample. PCoA was calculated using Bray-Curtis distances with a multivariate t-distribution. Ellipses represent an 80% confidence level. Color is indicative of cohort. (For interpretation of the references to colour in this figure legend, the reader is referred to the Web version of this article.)

To discover the effect of mixed herbs and the source of cheese localization on the metagenomic diversity and richness heatmap clustering and PCoA analyses were performed. According to the heatmap graph, bacterial species diversity and similarity of species composition among cheese samples were not significantly affected by mixing different herb species (Fig. 2). Although metagenomic analysis indicated three main clusters according to the PCoA graph, no significant difference was not observed based on localization among the clusters (Fig. 3).

## 4 Discussion

Here, we studied the bacterial compositions of the herby cheeses frequently consumed in the south-eastern regions of Turkey utilizing 16S metagenome analysis. Other than the different types of homemade cheeses there is only one report on herby cheese metagenome analysis [27]. Compared to that we utilized more samples collected from diverse with the identification of a much higher number of OTUs. Although a few samples have low Shannon and Simpson indices, average values determined as 3.03 ± 0.65 and 0.83 ± 0.08, respectively (Table 2). On the other hand, we identified 2.950 bacteria species 114 of them was present with an abundance of at least 0.5% in at least one sample (Supplementary Table 1). Therefore, the diversity of species is rich and evenness of bacterial composition is high in the samples. Additionally, no significant difference was observed between herby and non-herby cheeses (Sample 1, Sample 3, Sample 5 and Sample 16) in terms of both Shannon and Simpson indices. It was reported that the herbs used in cheese making provide the cheese with a characteristic appearance and aroma. Besides, antioxidant effects of the mixed herbs increase the shelf life of the cheese [28,29]. Cheese metagenome in terms of bacterial species diversity and richness is not affected by the mixing of the herbs. However, mixing different herbs during cheese making process may add aroma, appearance, and durability to the cheese.

In terms of localization, Sample 33 and Sample 35 samples collected from Cizre and Şırnak (Center) regions have higher Shannon and Simpson index values than the averages of other regions (3.532, 0.9332 and 3.965, 0.9347, respectively). Furthermore, no significant difference was observed between the Shannon and Simpson indices of cheese samples collected from other localizations (Table 3). Whereas, *Streptococcus parauberis*, which causes inflammation in the sheep udder mastitis [30], was determined as the dominant bacterial species (17.21%) in Sample 33 sample. Similarly, we detected that the metagenome of the Sample 35 sample contains *Bradyrhizobium* sp. PSBB068, a soil-dwelling bacterium [31], is at a highly abundant level 20.59%. Since the ripening process of the herby cheese takes place under the ground, bacteria may have passed from the soil to the cheese. Consequently, it can be concluded that no significant effect on cheese metagenome is observed according to the localizations where cheese manufactured (Table 3).

Shannon and Simpson indices were determined in previous metagenome studies on various fermented milk products. Shannon index values were found between 1.219 and 1.681 in kefir [21], 0.91–1.86 in Siciliano cheese [23], 0.6–1.61 in Feta cheese [24], 0.4–1.76 in Cheddar, provolone and Swiss cheeses [25], 0.69–4.57 in Kazakh cheese [26], while Simpson index values were between 0.5246 and 0.6686 in kefir [21] and in Siciliano cheese 0.39–0.79 [23]. Briefly, we observed that bacterial richness and evenness of herby cheese are higher than such ripened milk products as Siciliano, Feta, Cheddar, Swiss cheeses, and kefir. It is more similar to Kazakh cheese in terms of species diversity. Moreover, the Shannon index values of herby cheese were reported between 0.276 and 1.317 [27]. Notable differences between the Shannon index values of herby cheese reported here and previous study [27] might be caused in the laboratory processes of the samples and number of sequencing reads.

Firmicutes is the dominant taxon at the phylum level in all of the examined cheese samples except one (Fig. 1A). Additionally, *Lactobacillaceae* and *Streptococcaceae* are abundant families across the analyzed microbiomes (Fig. 1B). These findings are consistent with the previous reports performed on milk products such as herby cheese [27], mozzarella cheese [32], Iranian liqvan cheese [33], Galotyri-like fresh acid-curd cheese [34], Feta cheese [24], Siciliano cheese [23] and yogurt, kefir, nunu, koumiss [35]. Non-lactic bacterial families such as *Enterobacteriaceae, Moraxellaceae*, and *Pseudomonadaceae* were observed in some samples, but not at a significant level in all samples (Fig. 1B, Supplementary Table 1). Similarly, mixed herbs used in cheese making and the source of cheese localizations did not significantly affect the differentiation of cheese samples at the phylum and family levels. In addition, no different bacterial composition was observed at the phylum and family levels in non-herby cheese samples compared to herby cheeses.

At the species level, *Companilactobacillus ginsenosidimutans* was observed as the dominant species in 16 of 36 cheese samples (Fig. 2). Moreover, *C. ginsenosidimutans* is consist of the bacterial composition at varying rates in all samples (range 0.54–55.63%) except Sample 35 in which number of reads (8,239) was lower than other samples (Table 2, Supplementary Table 1). *C. ginsenosidimutans* was isolated for the first time from kimchi, a traditional Korean food manufactured by fermenting vegetables with probiotic lactic acid bacteria [36]. *C. ginsenosidimutans* is Gram-positive, facultatively anaerobic, non-motile, non-spore-forming, and rod-shaped lactic acid bacterial strain and can converts ginsenosides by β-glucosidase activity [37,38]. In addition to Kimchi, other foods with C*. ginsenosidimutans* were detected in pepper and *Brassica napobrassica* pickles [39]. While Sudagidan et al. (2021) analyzed Turkish herby cheese metagenome they did not detect *C. ginsenosidimutans* [27] Source of cheese samples and/or the depth of sequencing reads might be the reason. The other bacterial species belonging to the genus *Companilactobacillus* is *C. alimentarius* detected in 9 of 36 samples (range 0.5–10.48%). One of *C. alimentarius* included samples is sheepskin-ripened cheese, the other is a curd cheese, and 7 other herby cheese samples. Again, the localization source of cheeses where the cheese samples were collected is not found to be significant. It has been stated that *C. alimentarius*, a lactic acid bacterium, has an antagonistic effect on three different pathogenic bacteria species (*Staphylococcus aureus, Bacillus subtilis,* and P*seudomonas aeruginosa*) and has antifungal activity against certain pathogen molds [40,41]. Besides, probiotic properties of *C. alimentarius* were reported [42,43] as well as it was identified in Turkish surk cheese samples [44].

Another remarkable species reported here is the *Weissella jogaejeotgali* species which was detected in 15 cheese samples (range 0.52–15.14%) regardless of mixture of herb and source of localization (Fig. 2, Supplementary Table 1). *W. jogaejeotgali* was first isolated from traditionally fermented Korean jogae jeotgal (fermented clams). It is described as Gram-positive, non-motile, irregularly rod-shaped lactic acid bacterial strain [45]. Genome analysis has shown that *W. jogaejeotgali* potentially is involved in food fermentation, osmotic stress resistance, acid tolerance, adhesion to the mucosal layer for survival with its probiotic potential and a high survival rate during food fermentation traits [46]. To date, some of the Weisella species were detected in various dairy products such as *W. sagaensis* in Chinese yogurt [47], *W. thailandensis* in Mexican Cotija cheese [48], *W. hellenica* in mozzarella cheese [49], *W. halotolerans*, *W. viridescens* in Cheddar cheese [50], and *W. viridescens* in Romanian cheese [51]. However, to the best of our knowledge, *W. jogaejeotgali* was detected for the first time in a dairy product in this study.

Although abundance of *Levilactobacillus koreensis* (formerly known as *Lactobacillus koreensis*) is low we have identified it in four cheese samples (range 0.59–3.07%). Similar to *C. ginsenosidimutans*, *L. koreensis* was isolated from Kimchi, a traditional Korean food manufactured by fermenting vegetables, for the first time [52]. Here, we listed *L. koreensis* from a dairy product.

As expected, we also identified some of lactic acid bacteria species such as; *Lactobacillus delbrueckii*, *Tetragenococcus halophilus, Lactococcus raffinolactis, Ligilactobacillus acidipiscis, Marinilactibacillus* sp. 15R, *Lactiplantibacillus plantarum, Secundilactobacillus malefermentans, Lactococcus cremoris, Levilactobacillus brevis* and *Lacticaseibacillus manihotivorans*. The most observed three of these lactic acid bacteria are *L. delbrueckii* detected in 20 of the 36 samples (range 0.51–54.4%), *L. raffinolactis* detected in 10 (range 0.64–31.89%), and *T. halophilus* detected in 9 (range 0.86–31.47%).

Furthermore, metagenome analysis observed undesirable bacterial species in herby cheese samples. *Streptococcus parauberis* was the abundant one detected in 29 of 36 samples (range 0.74–37.55%). *S. parauberis* causes inflammation in the sheep udder mastitis [30], even became dominant in some cheese samples. Although not as common as *S. parauberis*, *S. suis, Mycoplasma agalactiae*, *S. pseudoporcinus, Staphylococcus aureus*, *S pyogenes*, and *Klebsiella pneumoniae* were identified in our analysis.

Additionally, some soil-dwelling bacterial species such as *Bradyrhizobium* sp. PSBB068, *Pseudomonas stutzeri, Bacillus thuringiensis, Acinetobacter johnsonii, Kitasatospora setae, Nocardioides dokdonensis* and, *Cupriavidus oxalaticus* were detected. *Bradyrhizobium* sp. PSBB068 was identified in 34 of 36 samples (range 0.55–26.09%) and even became dominant species in some samples. The ripening process of herby cheese takes place under the ground for six months. Therefore, the bacteria might pass from the soil to the cheese due to the untightly fitting lid of jars during ripening.

## 5 Conclusion

Şırnak province in Turkey is located in the region called “Fertile Crescent” where both the homeland and domestication center of the sheep, goats, cows, pigs, and wheat is known today [53]. Such factors as altitude, temperature, landforms, bedrock structure, precipitation regime, and wind greatly enrich the flora that is the source of the plant diversity included in the cheese and the grasses that the sheep feed during grazing. The detection of *C. ginsenosidimutans, W. jogaejeotgali* and *L. koreensis* in subjected cheese samples for the first time may be due to the fact that Şırnak province is located in a special geographical location and is fed with plants grown in this region. However, these bacteria have generally been observed in foods made in far eastern regions such as Korea and China. Turkey is geographically far from these countries. The main limitation of this study is the geographical area of the collected samples which is resemble a small part of the country. Therefore, it is interesting that common bacteria are observed in the food of regions that are so far apart from each other, that there is no record of these bacteria among these regions. Further high-throughput bacterial studies are needed to elucidate this situation.

It is assumed that the results of the study will play an important role in obtaining the geographical indications of the cheeses produced in Şırnak. Thus, with the increase in the brand value of the cheese produced, it is also possible to benefit from the other functions offered by the geographical indications specified as discrimination function, citation function, quality and warranty function, advertisement function, function of protecting traditional knowledge and cultural values, and the function of providing local development.

## Author contribution statement

Mustafa Rüstemoğlu: Conceived and designed the experiments; Performed the experiments; Analyzed and interpreted the data; Contributed reagents, materials, analysis tools or data; Wrote the paper.

Gazal Cengiz: Contributed reagents, materials, analysis tools or data; Wrote the paper.

Mehmet Emin Erkan; Murteza Hacızade; Analyzed and interpreted the data; Contributed reagents, materials, analysis tools or data.

## Funding statement

This work was supported by the Southeastern Anatolia Project Regional Development Administration (GAP).

## Data availability statement

Data included in article/supp. material/referenced in article.

## Declaration of interest’s statement

The authors declare no competing interests.
