# Supplementary material for: Bacterial metagenome profiling of hand-made herby cheese samples utilizing high-throughput sequencing to detect geographical indication and marketing potential
Source: Heliyon. 2023 Feb 9;9(2):e13334. doi: 10.1016/j.heliyon.2023.e13334 (PMC9950837; doi:10.1016/j.heliyon.2023.e13334)
Supplement: Multimedia component 1 [file mmc1.docx]

**16S NGS METAGENOM ANALİZ RAPORU**

**
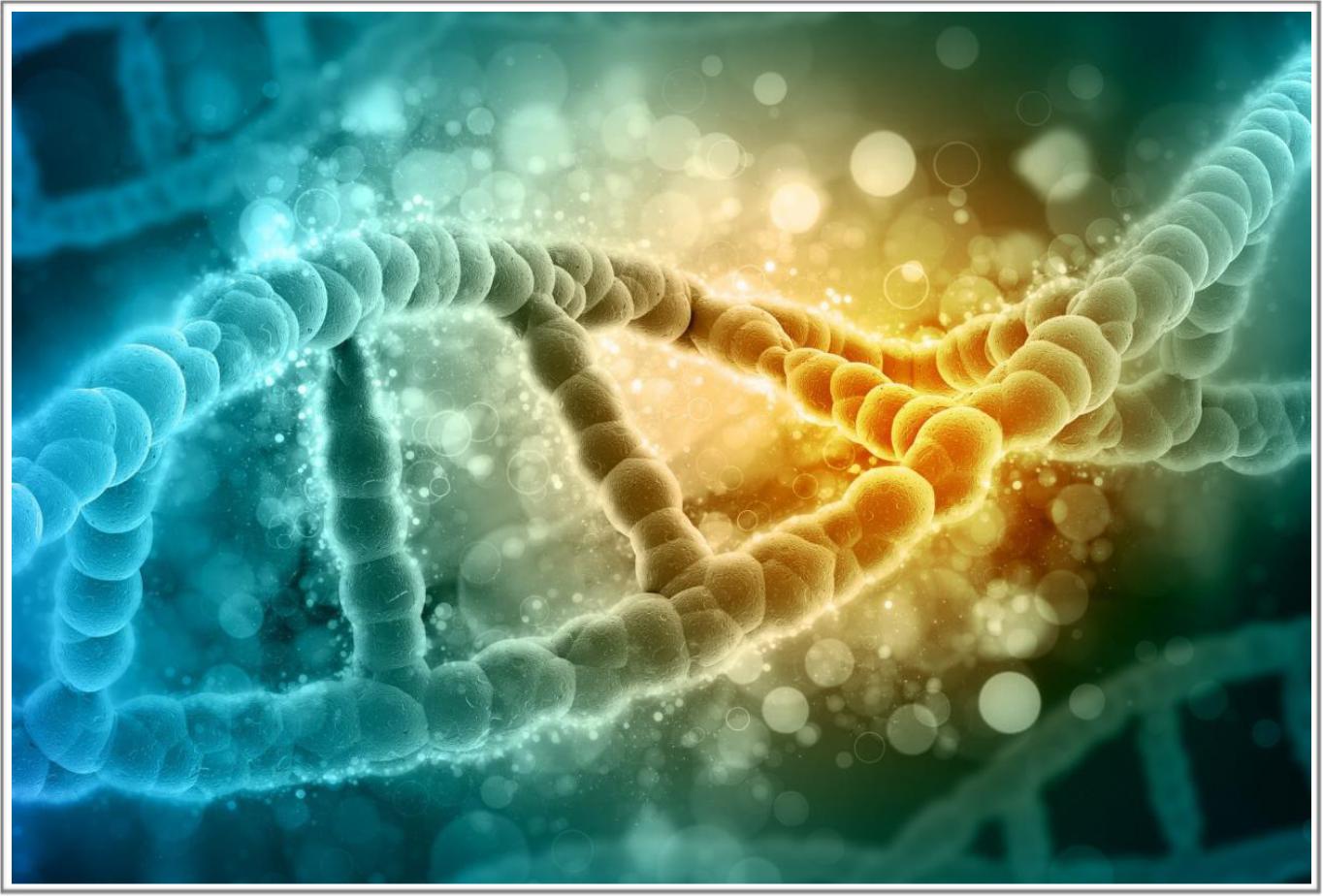
**

**2021**

# 1. Analiz Adımları

# 1.1 Örneklerden DNA izolasyonu

# Peynir örneklerinden genomik DNA izolasyonu Zymo Research firmasına ait “Quick-DNA ^TM^ Fungal/Bacterial Miniprep Kit, Cat. No.: D6005” kullanılarak yapılmıştır. İzole edilmiş DNA’nın miktar ve saflığı florometrik olarak Qubit ile tayin edilmiştir.

# 1.2 *16s rRNA* V3-V4 bölgesinin amplifikasyonu

# Tür tayininde kullanılacak olan *16s rRNA* genine ait V3-V4 bölgeleri üniversal 341F-805R primer dizileri ile SimpliAmp Thermal Cycler kullanılarak amplifiye edilmiştir. Kullanılan primer dizileri ve PCR koşulları aşağıda verilmiştir.

# 341F: CCTACGGGNGGCWGCAG

# 805R: GACTACHVGGGTATCTAATCC

# 95°C 10 dakika – initial denaturation (HS enzim kullanılmıştır)

# 35 döngü:

# — 95°C for 30 saniye - denaturation

# — 53-48°C for 30 saniye – annealing (touchdown PCR)

# — 72°C for 15 saniye – extension

# Sıcaklık 4°C’ye düşürülüp PCR tamamlanmıştır.

# 1.3 Kütüphane hazırlama ve dizileme işlemi

# 16s rRNA V3-V4 amplikon ürünleri için kütüphane hazırlama Illumina’nın “Nextera XT DNA Library Prep Kit, Cat. No.: FC-131-1096” ile index işlemi ise “TG Nextera XT Index Kit v2 Set A (96 Indices, 384 Samples), Cat. No.: TG-131-2001” ile yapılmıştır. PCR pürifikasyon işlemleri Beckman Coulter firmasına ait “AMPure XP beads” ile yapılmıştır. Dizileme işlemi Illumina’nın Miseq platformu ile paired-end (PE) 2x150 bazlık okumalar olarak yapılmıştır. Örnek başına minimum ≥ 50.000 okuma yapılmıştır.

# 1.4 Ham verinin biyoinformatik analizi

# Ham veri okumaları (FASTQ) QC kontrol yapılmış, trim edilmiş (gerekli görülürse) ve Kraken Metagenomik sistemi ile OTU sınıflarına ayrılmıştır. Kraken uygulaması, yüksek hassasiyet ve hızda kısa DNA sekanslarına taksonomik etiketler atar. Daha fazla bilgi için “Wood, D. E., & Salzberg, S. L. (2014). Kraken: ultrafast metagenomic sequence classification using exact alignments. Genome biology, 15(3), R46.” referanslı makaleye bakınız.

# 1.5 Raporlama

# Örneklerdeki bakteriyal dağılım ve çeşitlilik rapor ve görsel halinde sunulmuştur. Ayrıca, OTU sınıfları ayrı ayrı olarak .excel dosyaları formatında verilmiştir.

**2. Taksonomik Sınıflandırma Raporu**

## 2.1 Dizileme İstatistiği

| **Örnek adı** | **Okuma sayısı** | **Ortalama okuma uzunluğu** | **Sınıflanmış**  **okuma** |
| --- | --- | --- | --- |
| Pey1 | 7062 | 107.2 | 7062 / 100.00% |
| Pey2 | 61008 | 103.9 | 61008 / 100.00% |
| Pey3 | 47138 | 113.4 | 47138 / 100.00% |
| Pey4 | 34211 | 110.3 | 34211 / 100.00% |
| Pey5 | 39491 | 107.0 | 39491 / 100.00% |
| Pey6 | 36561 | 106.6 | 36561 / 100.00% |
| Pey8 | 22420 | 112.7 | 22420 / 100.00% |
| Pey9 | 34123 | 110.7 | 34123 / 100.00% |
| Pey10 | 27509 | 109.3 | 27509 / 100.00% |
| Pey11 | 29223 | 109.8 | 29223 / 100.00% |
| Pey12 | 34664 | 108.0 | 34664 / 100.00% |
| Pey13 | 42756 | 109.1 | 42756 / 100.00% |
| Pey14 | 13213 | 110.7 | 13213 / 100.00% |
| Pey15 | 35152 | 112.2 | 35152 / 100.00% |
| Pey16 | 20350 | 114.3 | 20350 / 100.00% |
| Pey17 | 45877 | 110.0 | 45877 / 100.00% |
| Pey18 | 180625 | 108.7 | 180625 / 100.00% |
| Pey19 | 56215 | 115.1 | 56215 / 100.00% |
| Pey20 | 30248 | 110.3 | 30248 / 100.00% |
| Pey21 | 24298 | 95.0 | 24298 / 100.00% |
| Pey22 | 59239 | 111.4 | 59239 / 100.00% |
| Pey23 | 47622 | 108.1 | 47622 / 100.00% |
| Pey24 | 52759 | 105.8 | 52759 / 100.00% |
| Pey25 | 76105 | 109.7 | 76105 / 100.00% |
| Pey26 | 75343 | 109.0 | 75343 / 100.00% |
| Pey27 | 12497 | 105.7 | 12497 / 100.00% |
| Pey28 | 41543 | 104.3 | 41543 / 100.00% |
| Pey29 | 16207 | 106.0 | 16207 / 100.00% |
| Pey30 | 34035 | 105.0 | 34035 / 100.00% |
| Pey31 | 49632 | 104.7 | 49632 / 100.00% |
| Pey32 | 25194 | 109.4 | 25194 / 100.00% |
| Pey33 | 21273 | 109.6 | 21273 / 100.00% |
| Pey34 | 7460 | 112.4 | 7460 / 100.00% |
| Pey35 | 8239 | 117.0 | 8239 / 100.00% |
| Pey36 | 50661 | 106.1 | 50661 / 100.00% |
| Pey37 | 56645 | 111.9 | 56645 / 100.00% |

**2.2 Taksonomi İstatistiği**

| **Tür Seviyesinde Çeşitlilik** | | |
| --- | --- | --- |
| **Örnek** | **Shannon Index (H) / (H / LN (N))** | **Simpson Index (1-D)** |
| Pey1 | 2.913 / 0.5434 | 0.8042 |
| Pey2 | 2.744 / 0.4573 | 0.7697 |
| Pey3 | 3.409 / 0.5854 | 0.8931 |
| Pey4 | 2.736 / 0.4481 | 0.775 |
| Pey5 | 4.81 / 0.7774 | 0.9792 |
| Pey6 | 2.612 / 0.4446 | 0.7435 |
| Pey8 | 2.84 / 0.5111 | 0.8458 |
| Pey9 | 3.034 / 0.5214 | 0.8394 |
| Pey10 | 2.931 / 0.5176 | 0.8282 |
| Pey11 | 3.11 / 0.5674 | 0.8693 |
| Pey12 | 2.001 / 0.3402 | 0.6756 |
| Pey13 | 2.18 / 0.3608 | 0.7066 |
| Pey14 | 3.534 / 0.6338 | 0.9205 |
| Pey15 | 2.794 / 0.4635 | 0.7977 |
| Pey16 | 3.593 / 0.5946 | 0.8965 |
| Pey17 | 2.861 / 0.4913 | 0.8354 |
| Pey18 | 2.533 / 0.4088 | 0.7726 |
| Pey19 | 2.13 / 0.3483 | 0.6814 |
| Pey20 | 3.35 / 0.5369 | 0.8448 |
| Pey21 | 2.625 / 0.4548 | 0.7967 |
| Pey22 | 2.368 / 0.3971 | 0.7767 |
| Pey23 | 1.831 / 0.3018 | 0.6569 |
| Pey24 | 2.609 / 0.4354 | 0.8273 |
| Pey25 | 3.41 / 0.5326 | 0.8995 |
| Pey26 | 3.161 / 0.5046 | 0.8222 |
| Pey27 | 3.433 / 0.5789 | 0.9044 |
| Pey28 | 2.788 / 0.4618 | 0.8371 |
| Pey29 | 3.334 / 0.5711 | 0.8964 |
| Pey30 | 2.847 / 0.4768 | 0.8384 |
| Pey31 | 3.753 / 0.6047 | 0.9328 |
| Pey32 | 1.812 / 0.3066 | 0.6355 |
| Pey33 | 3.532 / 0.6527 | 0.9332 |
| Pey34 | 3.921 / 0.651 | 0.9122 |
| Pey35 | 3.965 / 0.7208 | 0.9347 |
| Pey36 | 3.605 / 0.589 | 0.9117 |
| Pey37 | 4.104 / 0.6291 | 0.9409 |

***** örneklerdeki tür çeşitliliğini gösterir. Simpsons indeksi 0-1 arasında bir değer alır. 1 çeşitliliği, 0 ise çeşitlilik yok anlamına gelir. Shannon indeksi genellikle 1,5-3,5 arasında bir değer alır ve bu indeks arttıkça çeşitlilik de artar.

**2.3 Çeşitlilik Analizleri**

Tür seviyesinde,

- Çeşitlilik eğrisi (diversity curve),
- Principal coordinate analysis (PCoA) plot,
- Seyreltme eğrileri (rarefaction curves) analizleri yapılmış ve ilgili görseller ekte “plot” dosyası içerisinde verilmiştir.

***Çeşitlilik eğrisi***, örneklerdeki toplam OTU’ların minimum, ortalama ve maximum sayısını gösterir. Bu grafik, benzer ve ya farklı veri setleri arasındaki çeşitliliği değerlendirmek için kullanılır. Bu analiz, 2 örnek ve üzerine uygulanabilir.

***PCoA plot***, örnekler arasındaki benzerlik ve farklılıkları gösteren 2-boyutlu bir grafiktir. Bu analiz, 2 örnek ve üzerine uygulanabilir.

***Seyreltme eğrileri,*** ekolojik çalışmalarda OTU analizinde yaygın bir şekilde kullanılan bir tekniktir. Bu grafik, *n* NGS okumasındaki (X-ekseni) beklenen OTU (Y-ekseni) sayısını gösterir. Seyreltme eğrilerinin amacı, dizileme kapsamının, belirli bir örnekte bulunan toplam OTU sayısını belirlemede yeterince derin olup olmadığını göstermektir. Eğer bu eğri platoya ulaşırsa, örneklerde tür çeşitliliğinin olduğunu gösterir. Bu analiz, 3 örnek ve üzerine uygulanabilir.

**3. Tür Seviyesinde Taksonomik Dağılımlar**

**Not:** örneklerdeki bakteriyal dağılımın tamamı ekte .excel dosyası içerisinde verilmiş olup, aşağıda en yüksek okuma dizisi bulunan 10 türe ait tablo verilmiştir.

| **Pey1** | | |
| --- | --- | --- |
| **Taxa** | **Count** | **%** |
| Companilactobacillus ginsenosidimutans | 884 | 42.26 |
| Weissella jogaejeotgali | 150 | 7.17 |
| Streptococcus parauberis | 140 | 6.69 |
| Bradyrhizobium sp. PSBB068 | 90 | 4.3 |
| Staphylococcus cohnii | 83 | 3.97 |
| Lactococcus cremoris | 75 | 3.59 |
| Companilactobacillus alimentarius | 75 | 3.59 |
| Levilactobacillus brevis | 46 | 2.2 |
| Staphylococcus aureus | 36 | 1.72 |
| Lactiplantibacillus plantarum | 25 | 1.2 |

| **Pey2** | | |
| --- | --- | --- |
| **Taxa** | **Count** | **%** |
| Companilactobacillus ginsenosidimutans | 6405 | 45.26 |
| Streptococcus parauberis | 1978 | 13.98 |
| Weissella jogaejeotgali | 691 | 4.88 |
| Lactococcus cremoris | 464 | 3.28 |
| Lactiplantibacillus plantarum | 364 | 2.57 |
| Fructilactobacillus sanfranciscensis | 219 | 1.55 |
| Streptococcus pyogenes | 206 | 1.46 |
| Ligilactobacillus acidipiscis | 201 | 1.42 |
| Streptococcus suis | 198 | 1.4 |
| Staphylococcus aureus | 195 | 1.38 |

| **Pey3** | | |
| --- | --- | --- |
| **Taxa** | **Count** | **%** |
| Companilactobacillus ginsenosidimutans | 2201 | 28.13 |
| Lactiplantibacillus plantarum | 901 | 11.52 |
| Streptococcus parauberis | 537 | 6.86 |
| Levilactobacillus brevis | 500 | 6.39 |
| Companilactobacillus alimentarius | 293 | 3.74 |
| Lactococcus cremoris | 261 | 3.34 |
| Staphylococcus aureus | 249 | 3.18 |
| Fructilactobacillus sanfranciscensis | 141 | 1.8 |
| Lapidilactobacillus dextrinicus | 115 | 1.47 |
| Streptococcus pyogenes | 107 | 1.37 |

| **Pey4** | | |
| --- | --- | --- |
| **Taxa** | **Count** | **%** |
| Companilactobacillus ginsenosidimutans | 4302 | 44.22 |
| Streptococcus parauberis | 1315 | 13.52 |
| Ligilactobacillus acidipiscis | 886 | 9.11 |
| Lactiplantibacillus plantarum | 244 | 2.51 |
| Lactococcus cremoris | 241 | 2.48 |
| Bradyrhizobium sp. PSBB068 | 185 | 1.9 |
| Streptococcus suis | 143 | 1.47 |
| Cutibacterium acnes | 131 | 1.35 |
| Weissella jogaejeotgali | 130 | 1.34 |
| Fructilactobacillus sanfranciscensis | 121 | 1.24 |

| **Pey5** | | |
| --- | --- | --- |
| **Taxa** | **Count** | **%** |
| Lactiplantibacillus plantarum | 209 | 5.9 |
| Staphylococcus aureus | 207 | 5.84 |
| Bradyrhizobium sp. PSBB068 | 191 | 5.39 |
| Lactococcus cremoris | 174 | 4.91 |
| Enterococcus faecalis | 134 | 3.78 |
| Nocardioides dokdonensis | 119 | 3.36 |
| Salmonella enterica | 119 | 3.36 |
| Enterococcus faecium | 110 | 3.11 |
| Escherichia coli | 82 | 2.32 |
| Bacillus velezensis | 69 | 1.95 |

| **Pey6** | | |
| --- | --- | --- |
| **Taxa** | **Count** | **%** |
| Companilactobacillus ginsenosidimutans | 5184 | 48.88 |
| Bacillus thuringiensis | 1058 | 9.98 |
| Lactiplantibacillus plantarum | 515 | 4.86 |
| Streptococcus parauberis | 513 | 4.84 |
| Bradyrhizobium sp. PSBB068 | 277 | 2.61 |
| Enterococcus faecium | 272 | 2.56 |
| Lactococcus cremoris | 199 | 1.88 |
| Tetragenococcus halophilus | 172 | 1.62 |
| Fructilactobacillus sanfranciscensis | 161 | 1.52 |
| Ligilactobacillus acidipiscis | 125 | 1.18 |

| **Pey8** | | |
| --- | --- | --- |
| **Taxa** | **Count** | **%** |
| Lactococcus raffinolactis | 1637 | 31.89 |
| Streptococcus parauberis | 975 | 18.99 |
| Streptococcus suis | 474 | 9.23 |
| Bacillus thuringiensis | 303 | 5.9 |
| Lactiplantibacillus plantarum | 218 | 4.25 |
| Bradyrhizobium sp. PSBB068 | 127 | 2.47 |
| Streptococcus pyogenes | 107 | 2.08 |
| Streptococcus pseudoporcinus | 97 | 1.89 |
| Staphylococcus cohnii | 79 | 1.54 |
| Streptococcus thermophilus | 76 | 1.48 |

| **Pey9** | | |
| --- | --- | --- |
| **Taxa** | **Count** | **%** |
| Streptococcus parauberis | 3093 | 36.63 |
| Companilactobacillus ginsenosidimutans | 1020 | 12.08 |
| Lactococcus raffinolactis | 573 | 6.79 |
| Bacillus thuringiensis | 429 | 5.08 |
| Lactiplantibacillus plantarum | 267 | 3.16 |
| Streptococcus pyogenes | 263 | 3.11 |
| Secundilactobacillus malefermentans | 246 | 2.91 |
| Bradyrhizobium sp. PSBB068 | 199 | 2.36 |
| Levilactobacillus koreensis | 149 | 1.76 |
| Streptococcus thermophilus | 92 | 1.09 |

| **Pey10** | | |
| --- | --- | --- |
| **Taxa** | **Count** | **%** |
| Streptococcus parauberis | 2585 | 37.55 |
| Companilactobacillus ginsenosidimutans | 989 | 14.36 |
| Lactococcus raffinolactis | 407 | 5.91 |
| Streptococcus pyogenes | 315 | 4.58 |
| Secundilactobacillus malefermentans | 242 | 3.51 |
| Lactiplantibacillus plantarum | 220 | 3.2 |
| Bacillus thuringiensis | 196 | 2.85 |
| Levilactobacillus koreensis | 144 | 2.09 |
| Lactococcus cremoris | 92 | 1.34 |
| Streptococcus thermophilus | 67 | 0.97 |

| **Pey11** | | |
| --- | --- | --- |
| **Taxa** | **Count** | **%** |
| Streptococcus parauberis | 2174 | 31.08 |
| Lactobacillus delbrueckii | 1032 | 14.75 |
| Lacticaseibacillus manihotivorans | 353 | 5.05 |
| Levilactobacillus zymae | 324 | 4.63 |
| Companilactobacillus ginsenosidimutans | 307 | 4.39 |
| Lactiplantibacillus plantarum | 306 | 4.37 |
| Streptococcus pyogenes | 258 | 3.69 |
| Levilactobacillus brevis | 228 | 3.26 |
| Marinilactibacillus sp. 15R | 86 | 1.23 |
| Streptococcus thermophilus | 81 | 1.16 |

| **Pey12** | | |
| --- | --- | --- |
| **Taxa** | **Count** | **%** |
| Companilactobacillus ginsenosidimutans | 6201 | 48.44 |
| Tetragenococcus halophilus | 3799 | 29.68 |
| Fructilactobacillus sanfranciscensis | 304 | 2.37 |
| Lactococcus cremoris | 249 | 1.95 |
| Lactiplantibacillus plantarum | 233 | 1.82 |
| Streptococcus parauberis | 160 | 1.25 |
| Bradyrhizobium sp. PSBB068 | 83 | 0.65 |
| Salmonella enterica | 70 | 0.55 |
| Enterococcus faecalis | 59 | 0.46 |
| Escherichia coli | 56 | 0.44 |

| **Pey13** | | |
| --- | --- | --- |
| **Taxa** | **Count** | **%** |
| Companilactobacillus ginsenosidimutans | 7068 | 45.85 |
| Tetragenococcus halophilus | 4377 | 28.39 |
| Streptococcus parauberis | 462 | 3.0 |
| Lactiplantibacillus plantarum | 334 | 2.17 |
| Fructilactobacillus sanfranciscensis | 316 | 2.05 |
| Lactococcus cremoris | 301 | 1.95 |
| Bradyrhizobium sp. PSBB068 | 187 | 1.21 |
| Salmonella enterica | 106 | 0.69 |
| Escherichia coli | 85 | 0.55 |
| Klebsiella pneumoniae | 80 | 0.52 |

| **Pey14** | | |
| --- | --- | --- |
| **Taxa** | **Count** | **%** |
| Companilactobacillus ginsenosidimutans | 569 | 18.33 |
| Streptococcus parauberis | 515 | 16.59 |
| Lactiplantibacillus plantarum | 261 | 8.41 |
| Lactococcus raffinolactis | 183 | 5.89 |
| [Haemophilus] ducreyi | 126 | 4.06 |
| Polynucleobacter necessarius | 116 | 3.74 |
| Bradyrhizobium sp. PSBB068 | 110 | 3.54 |
| Streptococcus suis | 101 | 3.25 |
| Levilactobacillus suantsaii | 83 | 2.67 |
| Streptococcus pyogenes | 76 | 2.45 |

| **Pey15** | | |
| --- | --- | --- |
| **Taxa** | **Count** | **%** |
| Companilactobacillus ginsenosidimutans | 4088 | 38.47 |
| Lactobacillus delbrueckii | 2347 | 22.09 |
| Tetragenococcus halophilus | 532 | 5.01 |
| Ligilactobacillus acidipiscis | 325 | 3.06 |
| Lactiplantibacillus plantarum | 239 | 2.25 |
| Lactococcus cremoris | 213 | 2.0 |
| Enterococcus faecalis | 130 | 1.22 |
| Salmonella enterica | 109 | 1.03 |
| Staphylococcus aureus | 101 | 0.95 |
| Levilactobacillus brevis | 97 | 0.91 |

| **Pey16** | | |
| --- | --- | --- |
| **Taxa** | **Count** | **%** |
| Streptococcus suis | 1257 | 28.63 |
| Streptococcus parauberis | 319 | 7.26 |
| Streptococcus pseudoporcinus | 298 | 6.79 |
| Companilactobacillus ginsenosidimutans | 292 | 6.65 |
| Weissella jogaejeotgali | 216 | 4.92 |
| Bradyrhizobium sp. PSBB068 | 178 | 4.05 |
| Enterococcus faecalis | 114 | 2.6 |
| Streptococcus thermophilus | 96 | 2.19 |
| Streptococcus pyogenes | 83 | 1.89 |
| Lactococcus cremoris | 74 | 1.69 |

| **Pey17** | | |
| --- | --- | --- |
| **Taxa** | **Count** | **%** |
| Lactobacillus delbrueckii | 6101 | 37.01 |
| Companilactobacillus ginsenosidimutans | 1813 | 11.0 |
| Levilactobacillus brevis | 1110 | 6.73 |
| Tetragenococcus halophilus | 795 | 4.82 |
| Levilactobacillus zymae | 772 | 4.68 |
| Streptococcus thermophilus | 757 | 4.59 |
| Lacticaseibacillus manihotivorans | 696 | 4.22 |
| Lactiplantibacillus plantarum | 651 | 3.95 |
| Bradyrhizobium sp. PSBB068 | 204 | 1.24 |
| Ligilactobacillus acidipiscis | 188 | 1.14 |

| **Pey18** | | |
| --- | --- | --- |
| **Taxa** | **Count** | **%** |
| Lactobacillus delbrueckii | 25876 | 45.18 |
| Lactococcus raffinolactis | 4935 | 8.62 |
| Companilactobacillus ginsenosidimutans | 4920 | 8.59 |
| Streptococcus parauberis | 2558 | 4.47 |
| Weissella jogaejeotgali | 2544 | 4.44 |
| Streptococcus suis | 2418 | 4.22 |
| Companilactobacillus alimentarius | 1961 | 3.42 |
| Ligilactobacillus acidipiscis | 1306 | 2.28 |
| Marinilactibacillus sp. 15R | 1167 | 2.04 |
| Lactococcus cremoris | 686 | 1.2 |

| **Pey19** | | |
| --- | --- | --- |
| **Taxa** | **Count** | **%** |
| Lactobacillus delbrueckii | 16304 | 54.4 |
| Ligilactobacillus acidipiscis | 2739 | 9.14 |
| Companilactobacillus ginsenosidimutans | 2471 | 8.24 |
| Streptococcus parauberis | 2183 | 7.28 |
| Streptococcus thermophilus | 801 | 2.67 |
| Bacillus anthracis | 716 | 2.39 |
| Tetragenococcus halophilus | 621 | 2.07 |
| Staphylococcus aureus | 462 | 1.54 |
| Staphylococcus debuckii | 242 | 0.81 |
| Salinicola tamaricis | 126 | 0.42 |

| **Pey20** | | |
| --- | --- | --- |
| **Taxa** | **Count** | **%** |
| Companilactobacillus ginsenosidimutans | 2055 | 37.01 |
| Lactococcus cremoris | 464 | 8.36 |
| Ligilactobacillus acidipiscis | 382 | 6.88 |
| Pseudomonas stutzeri | 223 | 4.02 |
| Lactococcus lactis | 190 | 3.42 |
| Staphylococcus aureus | 168 | 3.03 |
| Bradyrhizobium sp. PSBB068 | 145 | 2.61 |
| Lactobacillus delbrueckii | 138 | 2.49 |
| Mycoplasma agalactiae | 117 | 2.11 |
| Salmonella enterica | 87 | 1.57 |

| **Pey21** | | |
| --- | --- | --- |
| **Taxa** | **Count** | **%** |
| Tetragenococcus halophilus | 2719 | 31.47 |
| Ligilactobacillus acidipiscis | 2699 | 31.23 |
| Staphylococcus aureus | 514 | 5.95 |
| Companilactobacillus ginsenosidimutans | 298 | 3.45 |
| Staphylococcus auricularis | 202 | 2.34 |
| Bradyrhizobium sp. PSBB068 | 163 | 1.89 |
| Rufibacter sp. DG15C | 161 | 1.86 |
| Carnobacterium sp. 17-4 | 86 | 1.0 |
| Staphylococcus haemolyticus | 74 | 0.86 |
| Bacillus thuringiensis | 68 | 0.79 |

| **Pey22** | | |
| --- | --- | --- |
| **Taxa** | **Count** | **%** |
| Companilactobacillus ginsenosidimutans | 8860 | 37.98 |
| Lactobacillus delbrueckii | 6024 | 25.83 |
| Streptococcus parauberis | 1977 | 8.48 |
| Lactococcus raffinolactis | 1410 | 6.04 |
| Lactiplantibacillus plantarum | 592 | 2.54 |
| Levilactobacillus zymae | 282 | 1.21 |
| Lactococcus cremoris | 252 | 1.08 |
| Fructilactobacillus sanfranciscensis | 227 | 0.97 |
| Clostridium perfringens | 225 | 0.96 |
| Lacticaseibacillus manihotivorans | 202 | 0.87 |

| **Pey23** | | |
| --- | --- | --- |
| **Taxa** | **Count** | **%** |
| Lactobacillus delbrueckii | 13212 | 52.1 |
| Companilactobacillus ginsenosidimutans | 6537 | 25.78 |
| Ligilactobacillus acidipiscis | 1454 | 5.73 |
| Streptococcus thermophilus | 1041 | 4.1 |
| Bradyrhizobium sp. PSBB068 | 196 | 0.77 |
| Staphylococcus aureus | 171 | 0.67 |
| Cupriavidus oxalaticus | 157 | 0.62 |
| Nocardioides dokdonensis | 102 | 0.4 |
| Bacillus anthracis | 96 | 0.38 |
| Enterococcus faecium | 84 | 0.33 |

| **Pey24** | | |
| --- | --- | --- |
| **Taxa** | **Count** | **%** |
| Streptococcus parauberis | 4374 | 25.01 |
| Companilactobacillus ginsenosidimutans | 4278 | 24.46 |
| Lactobacillus delbrueckii | 3814 | 21.81 |
| Staphylococcus cohnii | 553 | 3.16 |
| Lentilactobacillus parabuchneri | 349 | 2.0 |
| Streptococcus pyogenes | 259 | 1.48 |
| Bradyrhizobium sp. PSBB068 | 248 | 1.42 |
| Lactiplantibacillus plantarum | 241 | 1.38 |
| Levilactobacillus brevis | 210 | 1.2 |
| Streptococcus thermophilus | 202 | 1.16 |

| **Pey25** | | |
| --- | --- | --- |
| **Taxa** | **Count** | **%** |
| Streptococcus parauberis | 4043 | 21.39 |
| Companilactobacillus ginsenosidimutans | 3672 | 19.42 |
| Ligilactobacillus acidipiscis | 1727 | 9.14 |
| Lactiplantibacillus plantarum | 947 | 5.01 |
| Lactobacillus delbrueckii | 722 | 3.82 |
| Lactococcus raffinolactis | 692 | 3.66 |
| Marinilactibacillus sp. 15R | 621 | 3.28 |
| Streptococcus thermophilus | 437 | 2.31 |
| Streptococcus pyogenes | 422 | 2.23 |
| Levilactobacillus brevis | 379 | 2.0 |

| **Pey26** | | |
| --- | --- | --- |
| **Taxa** | **Count** | **%** |
| Lactobacillus delbrueckii | 6284 | 40.27 |
| Lactococcus raffinolactis | 1178 | 7.55 |
| Levilactobacillus brevis | 778 | 4.99 |
| Streptococcus thermophilus | 768 | 4.92 |
| Acinetobacter johnsonii | 515 | 3.3 |
| Lactiplantibacillus plantarum | 506 | 3.24 |
| Streptococcus parauberis | 475 | 3.04 |
| Lactococcus cremoris | 409 | 2.62 |
| Salmonella enterica | 177 | 1.13 |
| Companilactobacillus ginsenosidimutans | 172 | 1.1 |

| **Pey27** | | |
| --- | --- | --- |
| **Taxa** | **Count** | **%** |
| Companilactobacillus ginsenosidimutans | 706 | 19.91 |
| Weissella jogaejeotgali | 537 | 15.14 |
| Bradyrhizobium sp. PSBB068 | 520 | 14.66 |
| Lactobacillus delbrueckii | 294 | 8.29 |
| Streptococcus parauberis | 142 | 4.0 |
| Lactococcus cremoris | 97 | 2.74 |
| Companilactobacillus alimentarius | 90 | 2.54 |
| Marinilactibacillus sp. 15R | 84 | 2.37 |
| Staphylococcus aureus | 40 | 1.13 |
| Lactiplantibacillus plantarum | 39 | 1.1 |

| **Pey28** | | |
| --- | --- | --- |
| **Taxa** | **Count** | **%** |
| Companilactobacillus ginsenosidimutans | 4823 | 34.11 |
| Tetragenococcus halophilus | 2069 | 14.63 |
| Streptococcus parauberis | 1796 | 12.7 |
| Companilactobacillus alimentarius | 769 | 5.44 |
| Bradyrhizobium sp. PSBB068 | 755 | 5.34 |
| Weissella jogaejeotgali | 602 | 4.26 |
| Lactobacillus delbrueckii | 328 | 2.32 |
| Micromonospora sp. WMMC415 | 156 | 1.1 |
| Lactiplantibacillus plantarum | 139 | 0.98 |
| Fructilactobacillus sanfranciscensis | 137 | 0.97 |

| **Pey29** | | |
| --- | --- | --- |
| **Taxa** | **Count** | **%** |
| Lactobacillus delbrueckii | 1042 | 25.17 |
| Bradyrhizobium sp. PSBB068 | 481 | 11.62 |
| Mycoplasma agalactiae | 451 | 10.89 |
| Companilactobacillus ginsenosidimutans | 326 | 7.87 |
| Streptococcus parauberis | 315 | 7.61 |
| Streptococcus thermophilus | 105 | 2.54 |
| Weissella jogaejeotgali | 89 | 2.15 |
| Lactococcus raffinolactis | 72 | 1.74 |
| Lactococcus cremoris | 70 | 1.69 |
| Lactiplantibacillus plantarum | 63 | 1.52 |

| **Pey30** | | |
| --- | --- | --- |
| **Taxa** | **Count** | **%** |
| Streptococcus parauberis | 4375 | 36.22 |
| Companilactobacillus alimentarius | 1266 | 10.48 |
| Companilactobacillus ginsenosidimutans | 896 | 7.42 |
| Weissella jogaejeotgali | 846 | 7.0 |
| Bradyrhizobium sp. PSBB068 | 637 | 5.27 |
| Lactobacillus delbrueckii | 610 | 5.05 |
| Streptococcus thermophilus | 387 | 3.2 |
| Marinilactibacillus sp. 15R | 341 | 2.82 |
| Lactiplantibacillus plantarum | 314 | 2.6 |
| Streptococcus pyogenes | 281 | 2.33 |

| **Pey31** | | |
| --- | --- | --- |
| **Taxa** | **Count** | **%** |
| Companilactobacillus ginsenosidimutans | 1836 | 18.21 |
| Weissella jogaejeotgali | 1067 | 10.58 |
| Bradyrhizobium sp. PSBB068 | 928 | 9.2 |
| Streptococcus parauberis | 740 | 7.34 |
| Ligilactobacillus acidipiscis | 540 | 5.36 |
| Staphylococcus aureus | 416 | 4.13 |
| Streptococcus suis | 342 | 3.39 |
| Lactiplantibacillus plantarum | 335 | 3.32 |
| Lactococcus cremoris | 216 | 2.14 |
| Streptomyces sp. ICC1 | 202 | 2.0 |

| **Pey32** | | |
| --- | --- | --- |
| **Taxa** | **Count** | **%** |
| Companilactobacillus ginsenosidimutans | 7430 | 55.63 |
| Marinilactibacillus sp. 15R | 2981 | 22.32 |
| Bradyrhizobium sp. PSBB068 | 829 | 6.21 |
| Fructilactobacillus sanfranciscensis | 458 | 3.43 |
| Schleiferilactobacillus harbinensis | 87 | 0.65 |
| Levilactobacillus zymae | 84 | 0.63 |
| Cutibacterium acnes | 84 | 0.63 |
| Lactococcus cremoris | 68 | 0.51 |
| Tetragenococcus halophilus | 67 | 0.5 |
| Desulfosarcina widdelii | 43 | 0.32 |

| **Pey33** | | |
| --- | --- | --- |
| **Taxa** | **Count** | **%** |
| Streptococcus parauberis | 510 | 17.21 |
| Companilactobacillus ginsenosidimutans | 301 | 10.16 |
| Bradyrhizobium sp. PSBB068 | 297 | 10.02 |
| Levilactobacillus brevis | 215 | 7.26 |
| Lactococcus cremoris | 175 | 5.91 |
| Streptococcus thermophilus | 128 | 4.32 |
| Lactiplantibacillus plantarum | 103 | 3.48 |
| Staphylococcus aureus | 91 | 3.07 |
| Levilactobacillus koreensis | 91 | 3.07 |
| Lactococcus lactis | 84 | 2.83 |

| **Pey34** | | |
| --- | --- | --- |
| **Taxa** | **Count** | **%** |
| Bradyrhizobium sp. PSBB068 | 720 | 26.09 |
| Ligilactobacillus acidipiscis | 277 | 10.04 |
| Salmonella enterica | 149 | 5.4 |
| Escherichia coli | 112 | 4.06 |
| Enterococcus faecalis | 83 | 3.01 |
| Companilactobacillus ginsenosidimutans | 80 | 2.9 |
| Klebsiella pneumoniae | 75 | 2.72 |
| Brucella pseudogrignonensis | 65 | 2.36 |
| Streptococcus parauberis | 62 | 2.25 |
| Cutibacterium acnes | 43 | 1.56 |

| **Pey35** | | |
| --- | --- | --- |
| **Taxa** | **Count** | **%** |
| Bradyrhizobium sp. PSBB068 | 229 | 20.59 |
| Lactococcus cremoris | 95 | 8.54 |
| Staphylococcus aureus | 90 | 8.09 |
| Acinetobacter radioresistens | 76 | 6.83 |
| Lactococcus lactis | 28 | 2.52 |
| Kitasatospora setae | 24 | 2.16 |
| Enterococcus faecalis | 22 | 1.98 |
| Lactiplantibacillus plantarum | 21 | 1.89 |
| Escherichia coli | 16 | 1.44 |
| Streptococcus thermophilus | 16 | 1.44 |

| **Pey36** | | |
| --- | --- | --- |
| **Taxa** | **Count** | **%** |
| Companilactobacillus ginsenosidimutans | 1620 | 19.62 |
| Bradyrhizobium sp. PSBB068 | 1597 | 19.34 |
| Streptococcus parauberis | 463 | 5.61 |
| Lactiplantibacillus plantarum | 384 | 4.65 |
| Levilactobacillus suantsaii | 378 | 4.58 |
| Lactococcus cremoris | 338 | 4.09 |
| Ligilactobacillus acidipiscis | 232 | 2.81 |
| Staphylococcus aureus | 165 | 2.0 |
| Streptococcus pyogenes | 149 | 1.8 |
| Streptococcus thermophilus | 134 | 1.62 |

| **Pey37** | | |
| --- | --- | --- |
| **Taxa** | **Count** | **%** |
| Streptococcus suis | 1697 | 17.24 |
| Bradyrhizobium sp. PSBB068 | 1352 | 13.74 |
| Streptococcus pseudoporcinus | 388 | 3.94 |
| Weissella jogaejeotgali | 379 | 3.85 |
| Lactococcus cremoris | 345 | 3.51 |
| Companilactobacillus ginsenosidimutans | 321 | 3.26 |
| Cupriavidus oxalaticus | 313 | 3.18 |
| Lactiplantibacillus plantarum | 259 | 2.63 |
| Salmonella enterica | 233 | 2.37 |
| Escherichia coli | 230 | 2.34 |

**4. Diğer Taksonomik Dağılımlar**

**Superkingdom**

| **Pey1** | | |
| --- | --- | --- |
| **Taxa** | **Count** | **%** |
| Bacteria <bacteria> | 7062 | 100.0 |

| **Pey2** | | |
| --- | --- | --- |
| **Taxa** | **Count** | **%** |
| Bacteria <bacteria> | 61008 | 100.0 |

| **Pey3** | | |
| --- | --- | --- |
| **Taxa** | **Count** | **%** |
| Bacteria <bacteria> | 47138 | 100.0 |

| **Pey4** | | |
| --- | --- | --- |
| **Taxa** | **Count** | **%** |
| Bacteria <bacteria> | 34211 | 100.0 |

| **Pey5** | | |
| --- | --- | --- |
| **Taxa** | **Count** | **%** |
| Bacteria <bacteria> | 39491 | 100.0 |

| **Pey6** | | |
| --- | --- | --- |
| **Taxa** | **Count** | **%** |
| Bacteria <bacteria> | 36561 | 100.0 |

| **Pey8** | | |
| --- | --- | --- |
| **Taxa** | **Count** | **%** |
| Bacteria <bacteria> | 22420 | 100.0 |

| **Pey9** | | |
| --- | --- | --- |
| **Taxa** | **Count** | **%** |
| Bacteria <bacteria> | 34123 | 100.0 |

| **Pey10** | | |
| --- | --- | --- |
| **Taxa** | **Count** | **%** |
| Bacteria <bacteria> | 27509 | 100.0 |

| **Pey11** | | |
| --- | --- | --- |
| **Taxa** | **Count** | **%** |
| Bacteria <bacteria> | 29223 | 100.0 |

| **Pey12** | | |
| --- | --- | --- |
| **Taxa** | **Count** | **%** |
| Bacteria <bacteria> | 34664 | 100.0 |

| **Pey13** | | |
| --- | --- | --- |
| **Taxa** | **Count** | **%** |
| Bacteria <bacteria> | 42756 | 100.0 |

| **Pey14** | | |
| --- | --- | --- |
| **Taxa** | **Count** | **%** |
| Bacteria <bacteria> | 13213 | 100.0 |

| **Pey15** | | |
| --- | --- | --- |
| **Taxa** | **Count** | **%** |
| Bacteria <bacteria> | 35152 | 100.0 |

| **Pey16** | | |
| --- | --- | --- |
| **Taxa** | **Count** | **%** |
| Bacteria <bacteria> | 20350 | 100.0 |

| **Pey17** | | |
| --- | --- | --- |
| **Taxa** | **Count** | **%** |
| Bacteria <bacteria> | 45877 | 100.0 |

| **Pey18** | | |
| --- | --- | --- |
| **Taxa** | **Count** | **%** |
| Bacteria <bacteria> | 180625 | 100.0 |

| **Pey19** | | |
| --- | --- | --- |
| **Taxa** | **Count** | **%** |
| Bacteria <bacteria> | 56215 | 100.0 |

| **Pey20** | | |
| --- | --- | --- |
| **Taxa** | **Count** | **%** |
| Bacteria <bacteria> | 30248 | 100.0 |

| **Pey21** | | |
| --- | --- | --- |
| **Taxa** | **Count** | **%** |
| Bacteria <bacteria> | 24298 | 100.0 |

| **Pey22** | | |
| --- | --- | --- |
| **Taxa** | **Count** | **%** |
| Bacteria <bacteria> | 59239 | 100.0 |

| **Pey23** | | |
| --- | --- | --- |
| **Taxa** | **Count** | **%** |
| Bacteria <bacteria> | 47622 | 100.0 |

| **Pey24** | | |
| --- | --- | --- |
| **Taxa** | **Count** | **%** |
| Bacteria <bacteria> | 52759 | 100.0 |

| **Pey25** | | |
| --- | --- | --- |
| **Taxa** | **Count** | **%** |
| Bacteria <bacteria> | 76105 | 100.0 |

| **Pey26** | | |
| --- | --- | --- |
| **Taxa** | **Count** | **%** |
| Bacteria <bacteria> | 75343 | 100.0 |

| **Pey27** | | |
| --- | --- | --- |
| **Taxa** | **Count** | **%** |
| Bacteria <bacteria> | 12497 | 100.0 |

| **Pey28** | | |
| --- | --- | --- |
| **Taxa** | **Count** | **%** |
| Bacteria <bacteria> | 41543 | 100.0 |

| **Pey29** | | |
| --- | --- | --- |
| **Taxa** | **Count** | **%** |
| Bacteria <bacteria> | 16207 | 100.0 |

| **Pey30** | | |
| --- | --- | --- |
| **Taxa** | **Count** | **%** |
| Bacteria <bacteria> | 34035 | 100.0 |

| **Pey31** | | |
| --- | --- | --- |
| **Taxa** | **Count** | **%** |
| Bacteria <bacteria> | 49632 | 100.0 |

| **Pey32** | | |
| --- | --- | --- |
| **Taxa** | **Count** | **%** |
| Bacteria <bacteria> | 25194 | 100.0 |

| **Pey33** | | |
| --- | --- | --- |
| **Taxa** | **Count** | **%** |
| Bacteria <bacteria> | 21273 | 100.0 |

| **Pey34** | | |
| --- | --- | --- |
| **Taxa** | **Count** | **%** |
| Bacteria <bacteria> | 7460 | 100.0 |

| **Pey35** | | |
| --- | --- | --- |
| **Taxa** | **Count** | **%** |
| Bacteria <bacteria> | 8239 | 100.0 |

| **Pey36** | | |
| --- | --- | --- |
| **Taxa** | **Count** | **%** |
| Bacteria <bacteria> | 50661 | 100.0 |

| **Pey37** | | |
| --- | --- | --- |
| **Taxa** | **Count** | **%** |
| Bacteria <bacteria> | 56645 | 100.0 |

**Phylum**

| **Pey1** | | |
| --- | --- | --- |
| **Taxa** | **Count** | **%** |
| Firmicutes | 5932 | 90.88 |
| Proteobacteria | 518 | 7.94 |
| Actinobacteria <actinobacteria> | 37 | 0.57 |
| Bacteroidetes <Bacteroidetes> | 13 | 0.2 |
| Tenericutes | 10 | 0.15 |
| Planctomycetes <Planctomycetes> | 4 | 0.06 |
| Chloroflexi <Chloroflexi> | 3 | 0.05 |
| Spirochaetes <Spirochaetes> | 3 | 0.05 |
| Thermodesulfobacteria <phylum> | 2 | 0.03 |
| Candidatus Saccharibacteria | 1 | 0.02 |

| **Pey2** | | |
| --- | --- | --- |
| **Taxa** | **Count** | **%** |
| Firmicutes | 53623 | 95.42 |
| Proteobacteria | 2027 | 3.61 |
| Actinobacteria <actinobacteria> | 362 | 0.64 |
| Bacteroidetes <Bacteroidetes> | 61 | 0.11 |
| Tenericutes | 53 | 0.09 |
| Spirochaetes <Spirochaetes> | 21 | 0.04 |
| Chloroflexi <Chloroflexi> | 8 | 0.01 |
| Thermodesulfobacteria <phylum> | 8 | 0.01 |
| Fusobacteria <Fusobacteria> | 6 | 0.01 |
| Cyanobacteria | 5 | 0.01 |

| **Pey3** | | |
| --- | --- | --- |
| **Taxa** | **Count** | **%** |
| Firmicutes | 43512 | 98.31 |
| Proteobacteria | 504 | 1.14 |
| Actinobacteria <actinobacteria> | 127 | 0.29 |
| Tenericutes | 40 | 0.09 |
| Bacteroidetes <Bacteroidetes> | 30 | 0.07 |
| Thermodesulfobacteria <phylum> | 8 | 0.02 |
| Aquificae <phylum> | 7 | 0.02 |
| Spirochaetes <Spirochaetes> | 7 | 0.02 |
| Cyanobacteria | 6 | 0.01 |
| Chloroflexi <Chloroflexi> | 5 | 0.01 |

| **Pey4** | | |
| --- | --- | --- |
| **Taxa** | **Count** | **%** |
| Firmicutes | 29758 | 93.74 |
| Proteobacteria | 1492 | 4.7 |
| Actinobacteria <actinobacteria> | 387 | 1.22 |
| Bacteroidetes <Bacteroidetes> | 35 | 0.11 |
| Tenericutes | 22 | 0.07 |
| Chloroflexi <Chloroflexi> | 10 | 0.03 |
| Cyanobacteria | 10 | 0.03 |
| Spirochaetes <Spirochaetes> | 7 | 0.02 |
| Planctomycetes <Planctomycetes> | 5 | 0.02 |
| Thermodesulfobacteria <phylum> | 5 | 0.02 |

| **Pey5** | | |
| --- | --- | --- |
| **Taxa** | **Count** | **%** |
| Firmicutes | 30277 | 89.49 |
| Proteobacteria | 2979 | 8.81 |
| Actinobacteria <actinobacteria> | 377 | 1.11 |
| Bacteroidetes <Bacteroidetes> | 60 | 0.18 |
| Tenericutes | 54 | 0.16 |
| Chloroflexi <Chloroflexi> | 20 | 0.06 |
| Thermodesulfobacteria <phylum> | 20 | 0.06 |
| Spirochaetes <Spirochaetes> | 12 | 0.04 |
| Cyanobacteria | 8 | 0.02 |
| Candidatus Saccharibacteria | 5 | 0.01 |

| **Pey6** | | |
| --- | --- | --- |
| **Taxa** | **Count** | **%** |
| Firmicutes | 32306 | 96.38 |
| Proteobacteria | 937 | 2.8 |
| Actinobacteria <actinobacteria> | 178 | 0.53 |
| Tenericutes | 32 | 0.1 |
| Bacteroidetes <Bacteroidetes> | 19 | 0.06 |
| Aquificae <phylum> | 11 | 0.03 |
| Spirochaetes <Spirochaetes> | 9 | 0.03 |
| Chloroflexi <Chloroflexi> | 5 | 0.01 |
| Thermodesulfobacteria <phylum> | 5 | 0.01 |
| Cyanobacteria | 3 | 0.01 |

| **Pey8** | | |
| --- | --- | --- |
| **Taxa** | **Count** | **%** |
| Firmicutes | 20621 | 96.88 |
| Proteobacteria | 545 | 2.56 |
| Actinobacteria <actinobacteria> | 53 | 0.25 |
| Tenericutes | 26 | 0.12 |
| Bacteroidetes <Bacteroidetes> | 23 | 0.11 |
| Chloroflexi <Chloroflexi> | 6 | 0.03 |
| Planctomycetes <Planctomycetes> | 4 | 0.02 |
| Aquificae <phylum> | 2 | 0.01 |
| Spirochaetes <Spirochaetes> | 2 | 0.01 |
| Fusobacteria <Fusobacteria> | 1 | 0.0 |

| **Pey9** | | |
| --- | --- | --- |
| **Taxa** | **Count** | **%** |
| Firmicutes | 28505 | 96.85 |
| Proteobacteria | 546 | 1.86 |
| Bacteroidetes <Bacteroidetes> | 144 | 0.49 |
| Actinobacteria <actinobacteria> | 133 | 0.45 |
| Candidatus Saccharibacteria | 33 | 0.11 |
| Tenericutes | 25 | 0.08 |
| Chloroflexi <Chloroflexi> | 15 | 0.05 |
| Spirochaetes <Spirochaetes> | 7 | 0.02 |
| Aquificae <phylum> | 5 | 0.02 |
| Thermodesulfobacteria <phylum> | 5 | 0.02 |

| **Pey10** | | |
| --- | --- | --- |
| **Taxa** | **Count** | **%** |
| Firmicutes | 24119 | 96.83 |
| Proteobacteria | 539 | 2.16 |
| Actinobacteria <actinobacteria> | 92 | 0.37 |
| Bacteroidetes <Bacteroidetes> | 78 | 0.31 |
| Candidatus Saccharibacteria | 34 | 0.14 |
| Tenericutes | 22 | 0.09 |
| Chloroflexi <Chloroflexi> | 8 | 0.03 |
| Thermodesulfobacteria <phylum> | 7 | 0.03 |
| Spirochaetes <Spirochaetes> | 4 | 0.02 |
| Fusobacteria <Fusobacteria> | 2 | 0.01 |

| **Pey11** | | |
| --- | --- | --- |
| **Taxa** | **Count** | **%** |
| Firmicutes | 26075 | 98.36 |
| Actinobacteria <actinobacteria> | 181 | 0.68 |
| Proteobacteria | 178 | 0.67 |
| Tenericutes | 42 | 0.16 |
| Cyanobacteria | 14 | 0.05 |
| Chloroflexi <Chloroflexi> | 6 | 0.02 |
| Thermodesulfobacteria <phylum> | 6 | 0.02 |
| Fusobacteria <Fusobacteria> | 4 | 0.02 |
| Bacteroidetes <Bacteroidetes> | 3 | 0.01 |
| Candidatus Saccharibacteria | 1 | 0.0 |

| **Pey12** | | |
| --- | --- | --- |
| **Taxa** | **Count** | **%** |
| Firmicutes | 30014 | 93.89 |
| Proteobacteria | 1769 | 5.53 |
| Actinobacteria <actinobacteria> | 96 | 0.3 |
| Tenericutes | 29 | 0.09 |
| Planctomycetes <Planctomycetes> | 15 | 0.05 |
| Chloroflexi <Chloroflexi> | 12 | 0.04 |
| Bacteroidetes <Bacteroidetes> | 9 | 0.03 |
| Thermodesulfobacteria <phylum> | 8 | 0.03 |
| Candidatus Saccharibacteria | 4 | 0.01 |
| Spirochaetes <Spirochaetes> | 2 | 0.01 |

| **Pey13** | | |
| --- | --- | --- |
| **Taxa** | **Count** | **%** |
| Firmicutes | 35614 | 90.28 |
| Proteobacteria | 3534 | 8.96 |
| Actinobacteria <actinobacteria> | 191 | 0.48 |
| Tenericutes | 30 | 0.08 |
| Bacteroidetes <Bacteroidetes> | 25 | 0.06 |
| Thermodesulfobacteria <phylum> | 14 | 0.04 |
| Chloroflexi <Chloroflexi> | 13 | 0.03 |
| Spirochaetes <Spirochaetes> | 8 | 0.02 |
| Cyanobacteria | 4 | 0.01 |
| Candidatus Saccharibacteria | 3 | 0.01 |

| **Pey14** | | |
| --- | --- | --- |
| **Taxa** | **Count** | **%** |
| Firmicutes | 11189 | 92.96 |
| Proteobacteria | 687 | 5.71 |
| Actinobacteria <actinobacteria> | 92 | 0.76 |
| Bacteroidetes <Bacteroidetes> | 25 | 0.21 |
| Planctomycetes <Planctomycetes> | 20 | 0.17 |
| Tenericutes | 9 | 0.07 |
| Cyanobacteria | 4 | 0.03 |
| Candidatus Saccharibacteria | 2 | 0.02 |
| Spirochaetes <Spirochaetes> | 2 | 0.02 |
| Thermodesulfobacteria <phylum> | 2 | 0.02 |

| **Pey15** | | |
| --- | --- | --- |
| **Taxa** | **Count** | **%** |
| Firmicutes | 29557 | 91.44 |
| Proteobacteria | 2293 | 7.09 |
| Actinobacteria <actinobacteria> | 263 | 0.81 |
| Bacteroidetes <Bacteroidetes> | 136 | 0.42 |
| Tenericutes | 32 | 0.1 |
| Chloroflexi <Chloroflexi> | 10 | 0.03 |
| Thermodesulfobacteria <phylum> | 10 | 0.03 |
| Planctomycetes <Planctomycetes> | 7 | 0.02 |
| Spirochaetes <Spirochaetes> | 5 | 0.02 |
| Cyanobacteria | 4 | 0.01 |

| **Pey16** | | |
| --- | --- | --- |
| **Taxa** | **Count** | **%** |
| Firmicutes | 15977 | 84.05 |
| Proteobacteria | 2704 | 14.22 |
| Actinobacteria <actinobacteria> | 158 | 0.83 |
| Bacteroidetes <Bacteroidetes> | 125 | 0.66 |
| Tenericutes | 12 | 0.06 |
| Spirochaetes <Spirochaetes> | 7 | 0.04 |
| Aquificae <phylum> | 6 | 0.03 |
| Chloroflexi <Chloroflexi> | 5 | 0.03 |
| Cyanobacteria | 3 | 0.02 |
| Thermodesulfobacteria <phylum> | 3 | 0.02 |

| **Pey17** | | |
| --- | --- | --- |
| **Taxa** | **Count** | **%** |
| Firmicutes | 41086 | 98.09 |
| Proteobacteria | 459 | 1.1 |
| Actinobacteria <actinobacteria> | 223 | 0.53 |
| Tenericutes | 48 | 0.11 |
| Spirochaetes <Spirochaetes> | 16 | 0.04 |
| Bacteroidetes <Bacteroidetes> | 14 | 0.03 |
| Chloroflexi <Chloroflexi> | 10 | 0.02 |
| Thermodesulfobacteria <phylum> | 6 | 0.01 |
| Planctomycetes <Planctomycetes> | 5 | 0.01 |
| Fusobacteria <Fusobacteria> | 4 | 0.01 |

| **Pey18** | | |
| --- | --- | --- |
| **Taxa** | **Count** | **%** |
| Firmicutes | 168377 | 98.87 |
| Proteobacteria | 1352 | 0.79 |
| Actinobacteria <actinobacteria> | 232 | 0.14 |
| Tenericutes | 132 | 0.08 |
| Bacteroidetes <Bacteroidetes> | 64 | 0.04 |
| Chloroflexi <Chloroflexi> | 45 | 0.03 |
| Planctomycetes <Planctomycetes> | 21 | 0.01 |
| Thermodesulfobacteria <phylum> | 15 | 0.01 |
| Candidatus Saccharibacteria | 13 | 0.01 |
| Aquificae <phylum> | 12 | 0.01 |

| **Pey19** | | |
| --- | --- | --- |
| **Taxa** | **Count** | **%** |
| Firmicutes | 49537 | 93.78 |
| Proteobacteria | 2043 | 3.87 |
| Actinobacteria <actinobacteria> | 915 | 1.73 |
| Tenericutes | 218 | 0.41 |
| Bacteroidetes <Bacteroidetes> | 48 | 0.09 |
| Planctomycetes <Planctomycetes> | 16 | 0.03 |
| Chloroflexi <Chloroflexi> | 9 | 0.02 |
| Cyanobacteria | 9 | 0.02 |
| Thermodesulfobacteria <phylum> | 7 | 0.01 |
| Fusobacteria <Fusobacteria> | 4 | 0.01 |

| **Pey20** | | |
| --- | --- | --- |
| **Taxa** | **Count** | **%** |
| Firmicutes | 25068 | 87.24 |
| Proteobacteria | 2928 | 10.19 |
| Tenericutes | 378 | 1.32 |
| Actinobacteria <actinobacteria> | 273 | 0.95 |
| Bacteroidetes <Bacteroidetes> | 35 | 0.12 |
| Spirochaetes <Spirochaetes> | 13 | 0.05 |
| Cyanobacteria | 10 | 0.03 |
| Chloroflexi <Chloroflexi> | 7 | 0.02 |
| Planctomycetes <Planctomycetes> | 6 | 0.02 |
| Thermodesulfobacteria <phylum> | 5 | 0.02 |

| **Pey21** | | |
| --- | --- | --- |
| **Taxa** | **Count** | **%** |
| Firmicutes | 17149 | 93.12 |
| Proteobacteria | 743 | 4.03 |
| Bacteroidetes <Bacteroidetes> | 224 | 1.22 |
| Actinobacteria <actinobacteria> | 207 | 1.12 |
| Tenericutes | 40 | 0.22 |
| Spirochaetes <Spirochaetes> | 13 | 0.07 |
| Chloroflexi <Chloroflexi> | 10 | 0.05 |
| Thermodesulfobacteria <phylum> | 7 | 0.04 |
| Chlorobi | 6 | 0.03 |
| Deinococcus-Thermus | 4 | 0.02 |

| **Pey22** | | |
| --- | --- | --- |
| **Taxa** | **Count** | **%** |
| Firmicutes | 54659 | 97.93 |
| Proteobacteria | 720 | 1.29 |
| Actinobacteria <actinobacteria> | 233 | 0.42 |
| Tenericutes | 73 | 0.13 |
| Bacteroidetes <Bacteroidetes> | 44 | 0.08 |
| Planctomycetes <Planctomycetes> | 26 | 0.05 |
| Spirochaetes <Spirochaetes> | 17 | 0.03 |
| Chloroflexi <Chloroflexi> | 12 | 0.02 |
| Thermodesulfobacteria <phylum> | 9 | 0.02 |
| Aquificae <phylum> | 8 | 0.01 |

| **Pey23** | | |
| --- | --- | --- |
| **Taxa** | **Count** | **%** |
| Firmicutes | 41980 | 94.54 |
| Proteobacteria | 1833 | 4.13 |
| Actinobacteria <actinobacteria> | 383 | 0.86 |
| Bacteroidetes <Bacteroidetes> | 52 | 0.12 |
| Tenericutes | 49 | 0.11 |
| Planctomycetes <Planctomycetes> | 45 | 0.1 |
| Cyanobacteria | 14 | 0.03 |
| Chloroflexi <Chloroflexi> | 12 | 0.03 |
| Aquificae <phylum> | 10 | 0.02 |
| Spirochaetes <Spirochaetes> | 10 | 0.02 |

| **Pey24** | | |
| --- | --- | --- |
| **Taxa** | **Count** | **%** |
| Firmicutes | 47146 | 96.85 |
| Proteobacteria | 639 | 1.31 |
| Actinobacteria <actinobacteria> | 435 | 0.89 |
| Tenericutes | 317 | 0.65 |
| Bacteroidetes <Bacteroidetes> | 62 | 0.13 |
| Chloroflexi <Chloroflexi> | 16 | 0.03 |
| Planctomycetes <Planctomycetes> | 14 | 0.03 |
| Aquificae <phylum> | 13 | 0.03 |
| Spirochaetes <Spirochaetes> | 13 | 0.03 |
| Fusobacteria <Fusobacteria> | 9 | 0.02 |

| **Pey25** | | |
| --- | --- | --- |
| **Taxa** | **Count** | **%** |
| Firmicutes | 61890 | 88.32 |
| Proteobacteria | 7320 | 10.45 |
| Actinobacteria <actinobacteria> | 645 | 0.92 |
| Bacteroidetes <Bacteroidetes> | 54 | 0.08 |
| Tenericutes | 50 | 0.07 |
| Thermodesulfobacteria <phylum> | 20 | 0.03 |
| Chloroflexi <Chloroflexi> | 17 | 0.02 |
| Spirochaetes <Spirochaetes> | 15 | 0.02 |
| Cyanobacteria | 14 | 0.02 |
| Planctomycetes <Planctomycetes> | 12 | 0.02 |

| **Pey26** | | |
| --- | --- | --- |
| **Taxa** | **Count** | **%** |
| Firmicutes | 63356 | 89.59 |
| Proteobacteria | 6835 | 9.67 |
| Actinobacteria <actinobacteria> | 308 | 0.44 |
| Bacteroidetes <Bacteroidetes> | 78 | 0.11 |
| Tenericutes | 74 | 0.1 |
| Chloroflexi <Chloroflexi> | 22 | 0.03 |
| Aquificae <phylum> | 7 | 0.01 |
| Thermodesulfobacteria <phylum> | 7 | 0.01 |
| Cyanobacteria | 6 | 0.01 |
| Candidatus Saccharibacteria | 5 | 0.01 |

| **Pey27** | | |
| --- | --- | --- |
| **Taxa** | **Count** | **%** |
| Firmicutes | 9841 | 85.47 |
| Proteobacteria | 1452 | 12.61 |
| Actinobacteria <actinobacteria> | 102 | 0.89 |
| Bacteroidetes <Bacteroidetes> | 82 | 0.71 |
| Tenericutes | 14 | 0.12 |
| Planctomycetes <Planctomycetes> | 5 | 0.04 |
| Chloroflexi <Chloroflexi> | 4 | 0.03 |
| Fusobacteria <Fusobacteria> | 3 | 0.03 |
| Spirochaetes <Spirochaetes> | 3 | 0.03 |
| Cyanobacteria | 2 | 0.02 |

| **Pey28** | | |
| --- | --- | --- |
| **Taxa** | **Count** | **%** |
| Firmicutes | 35257 | 92.12 |
| Proteobacteria | 2333 | 6.1 |
| Actinobacteria <actinobacteria> | 542 | 1.42 |
| Tenericutes | 56 | 0.15 |
| Bacteroidetes <Bacteroidetes> | 24 | 0.06 |
| Chloroflexi <Chloroflexi> | 16 | 0.04 |
| Planctomycetes <Planctomycetes> | 8 | 0.02 |
| Spirochaetes <Spirochaetes> | 6 | 0.02 |
| Thermodesulfobacteria <phylum> | 6 | 0.02 |
| Fusobacteria <Fusobacteria> | 5 | 0.01 |

| **Pey29** | | |
| --- | --- | --- |
| **Taxa** | **Count** | **%** |
| Firmicutes | 11701 | 78.82 |
| Tenericutes | 1526 | 10.28 |
| Proteobacteria | 1365 | 9.19 |
| Actinobacteria <actinobacteria> | 208 | 1.4 |
| Deinococcus-Thermus | 14 | 0.09 |
| Bacteroidetes <Bacteroidetes> | 9 | 0.06 |
| Fusobacteria <Fusobacteria> | 7 | 0.05 |
| Planctomycetes <Planctomycetes> | 6 | 0.04 |
| Spirochaetes <Spirochaetes> | 4 | 0.03 |
| Chlorobi | 1 | 0.01 |

| **Pey30** | | |
| --- | --- | --- |
| **Taxa** | **Count** | **%** |
| Firmicutes | 29131 | 94.33 |
| Proteobacteria | 1244 | 4.03 |
| Actinobacteria <actinobacteria> | 292 | 0.95 |
| Tenericutes | 141 | 0.46 |
| Bacteroidetes <Bacteroidetes> | 27 | 0.09 |
| Chloroflexi <Chloroflexi> | 12 | 0.04 |
| Planctomycetes <Planctomycetes> | 8 | 0.03 |
| Thermodesulfobacteria <phylum> | 7 | 0.02 |
| Cyanobacteria | 5 | 0.02 |
| Spirochaetes <Spirochaetes> | 5 | 0.02 |

| **Pey31** | | |
| --- | --- | --- |
| **Taxa** | **Count** | **%** |
| Firmicutes | 41936 | 94.26 |
| Proteobacteria | 1729 | 3.89 |
| Actinobacteria <actinobacteria> | 628 | 1.41 |
| Cyanobacteria | 64 | 0.14 |
| Tenericutes | 52 | 0.12 |
| Bacteroidetes <Bacteroidetes> | 26 | 0.06 |
| Spirochaetes <Spirochaetes> | 11 | 0.02 |
| Fusobacteria <Fusobacteria> | 10 | 0.02 |
| Thermodesulfobacteria <phylum> | 10 | 0.02 |
| Candidatus Saccharibacteria | 7 | 0.02 |

| **Pey32** | | |
| --- | --- | --- |
| **Taxa** | **Count** | **%** |
| Firmicutes | 21313 | 92.85 |
| Proteobacteria | 1295 | 5.64 |
| Actinobacteria <actinobacteria> | 259 | 1.13 |
| Bacteroidetes <Bacteroidetes> | 30 | 0.13 |
| Tenericutes | 18 | 0.08 |
| Spirochaetes <Spirochaetes> | 13 | 0.06 |
| Fusobacteria <Fusobacteria> | 10 | 0.04 |
| Chloroflexi <Chloroflexi> | 5 | 0.02 |
| Cyanobacteria | 3 | 0.01 |
| Fibrobacteres <Fibrobacteres> | 2 | 0.01 |

| **Pey33** | | |
| --- | --- | --- |
| **Taxa** | **Count** | **%** |
| Firmicutes | 19629 | 96.85 |
| Proteobacteria | 489 | 2.41 |
| Actinobacteria <actinobacteria> | 117 | 0.58 |
| Bacteroidetes <Bacteroidetes> | 12 | 0.06 |
| Tenericutes | 9 | 0.04 |
| Spirochaetes <Spirochaetes> | 3 | 0.01 |
| Thermodesulfobacteria <phylum> | 2 | 0.01 |
| Candidatus Saccharibacteria | 1 | 0.0 |
| Verrucomicrobia | 1 | 0.0 |
| Chloroflexi <Chloroflexi> | 1 | 0.0 |

| **Pey34** | | |
| --- | --- | --- |
| **Taxa** | **Count** | **%** |
| Proteobacteria | 4390 | 67.79 |
| Firmicutes | 1647 | 25.43 |
| Actinobacteria <actinobacteria> | 226 | 3.49 |
| Bacteroidetes <Bacteroidetes> | 108 | 1.67 |
| Cyanobacteria | 55 | 0.85 |
| Spirochaetes <Spirochaetes> | 29 | 0.45 |
| Fusobacteria <Fusobacteria> | 8 | 0.12 |
| Planctomycetes <Planctomycetes> | 4 | 0.06 |
| Tenericutes | 2 | 0.03 |
| Deinococcus-Thermus | 2 | 0.03 |

| **Pey35** | | |
| --- | --- | --- |
| **Taxa** | **Count** | **%** |
| Firmicutes | 6885 | 91.28 |
| Proteobacteria | 511 | 6.77 |
| Actinobacteria <actinobacteria> | 113 | 1.5 |
| Bacteroidetes <Bacteroidetes> | 15 | 0.2 |
| Tenericutes | 7 | 0.09 |
| Spirochaetes <Spirochaetes> | 4 | 0.05 |
| Cyanobacteria | 3 | 0.04 |
| Fusobacteria <Fusobacteria> | 2 | 0.03 |
| Planctomycetes <Planctomycetes> | 1 | 0.01 |
| Gemmatimonadetes <phylum> | 1 | 0.01 |

| **Pey36** | | |
| --- | --- | --- |
| **Taxa** | **Count** | **%** |
| Firmicutes | 44483 | 93.94 |
| Proteobacteria | 2268 | 4.79 |
| Actinobacteria <actinobacteria> | 459 | 0.97 |
| Bacteroidetes <Bacteroidetes> | 50 | 0.11 |
| Tenericutes | 35 | 0.07 |
| Cyanobacteria | 16 | 0.03 |
| Chloroflexi <Chloroflexi> | 8 | 0.02 |
| Spirochaetes <Spirochaetes> | 8 | 0.02 |
| Thermodesulfobacteria <phylum> | 7 | 0.01 |
| Planctomycetes <Planctomycetes> | 5 | 0.01 |

| **Pey37** | | |
| --- | --- | --- |
| **Taxa** | **Count** | **%** |
| Firmicutes | 42668 | 79.69 |
| Proteobacteria | 10400 | 19.42 |
| Actinobacteria <actinobacteria> | 346 | 0.65 |
| Bacteroidetes <Bacteroidetes> | 30 | 0.06 |
| Tenericutes | 28 | 0.05 |
| Spirochaetes <Spirochaetes> | 19 | 0.04 |
| Thermodesulfobacteria <phylum> | 12 | 0.02 |
| Cyanobacteria | 11 | 0.02 |
| Chloroflexi <Chloroflexi> | 10 | 0.02 |
| Fusobacteria <Fusobacteria> | 5 | 0.01 |

**Class**

| **Pey1** | | |
| --- | --- | --- |
| **Taxa** | **Count** | **%** |
| Bacilli | 5773 | 90.13 |
| Gammaproteobacteria | 303 | 4.73 |
| Alphaproteobacteria | 129 | 2.01 |
| Betaproteobacteria | 67 | 1.05 |
| Clostridia | 48 | 0.75 |
| Actinomycetia | 32 | 0.5 |
| Mollicutes | 10 | 0.16 |
| Erysipelotrichia | 7 | 0.11 |
| Bacteroidia | 5 | 0.08 |
| Planctomycetia | 4 | 0.06 |

| **Pey2** | | |
| --- | --- | --- |
| **Taxa** | **Count** | **%** |
| Bacilli | 52541 | 95.22 |
| Gammaproteobacteria | 1637 | 2.97 |
| Actinomycetia | 352 | 0.64 |
| Alphaproteobacteria | 226 | 0.41 |
| Betaproteobacteria | 111 | 0.2 |
| Clostridia | 76 | 0.14 |
| Mollicutes | 52 | 0.09 |
| Bacteroidia | 33 | 0.06 |
| Spirochaetia | 21 | 0.04 |
| Deltaproteobacteria | 20 | 0.04 |

| **Pey3** | | |
| --- | --- | --- |
| **Taxa** | **Count** | **%** |
| Bacilli | 43004 | 98.21 |
| Gammaproteobacteria | 317 | 0.72 |
| Actinomycetia | 125 | 0.29 |
| Alphaproteobacteria | 117 | 0.27 |
| Betaproteobacteria | 54 | 0.12 |
| Mollicutes | 40 | 0.09 |
| Clostridia | 30 | 0.07 |
| Bacteroidia | 11 | 0.03 |
| Flavobacteriia | 11 | 0.03 |
| Epsilonproteobacteria | 9 | 0.02 |

| **Pey4** | | |
| --- | --- | --- |
| **Taxa** | **Count** | **%** |
| Bacilli | 29281 | 93.65 |
| Gammaproteobacteria | 1134 | 3.63 |
| Actinomycetia | 377 | 1.21 |
| Alphaproteobacteria | 246 | 0.79 |
| Betaproteobacteria | 48 | 0.15 |
| Clostridia | 32 | 0.1 |
| Mollicutes | 22 | 0.07 |
| Deltaproteobacteria | 20 | 0.06 |
| Flavobacteriia | 20 | 0.06 |
| Epsilonproteobacteria | 10 | 0.03 |

| **Pey5** | | |
| --- | --- | --- |
| **Taxa** | **Count** | **%** |
| Bacilli | 29510 | 89.09 |
| Gammaproteobacteria | 2551 | 7.7 |
| Actinomycetia | 369 | 1.11 |
| Alphaproteobacteria | 288 | 0.87 |
| Betaproteobacteria | 74 | 0.22 |
| Clostridia | 65 | 0.2 |
| Mollicutes | 54 | 0.16 |
| Bacteroidia | 31 | 0.09 |
| Erysipelotrichia | 26 | 0.08 |
| Thermodesulfobacteria <class> | 20 | 0.06 |

| **Pey6** | | |
| --- | --- | --- |
| **Taxa** | **Count** | **%** |
| Bacilli | 31687 | 96.25 |
| Alphaproteobacteria | 493 | 1.5 |
| Gammaproteobacteria | 364 | 1.11 |
| Actinomycetia | 176 | 0.53 |
| Betaproteobacteria | 58 | 0.18 |
| Mollicutes | 32 | 0.1 |
| Clostridia | 28 | 0.09 |
| Aquificae <class> | 11 | 0.03 |
| Flavobacteriia | 11 | 0.03 |
| Spirochaetia | 9 | 0.03 |

| **Pey8** | | |
| --- | --- | --- |
| **Taxa** | **Count** | **%** |
| Bacilli | 20334 | 96.81 |
| Gammaproteobacteria | 319 | 1.52 |
| Alphaproteobacteria | 186 | 0.89 |
| Actinomycetia | 51 | 0.24 |
| Betaproteobacteria | 29 | 0.14 |
| Mollicutes | 26 | 0.12 |
| Clostridia | 16 | 0.08 |
| Flavobacteriia | 13 | 0.06 |
| Bacteroidia | 6 | 0.03 |
| Dehalococcoidia | 5 | 0.02 |

| **Pey9** | | |
| --- | --- | --- |
| **Taxa** | **Count** | **%** |
| Bacilli | 28059 | 96.74 |
| Alphaproteobacteria | 238 | 0.82 |
| Gammaproteobacteria | 213 | 0.73 |
| Actinomycetia | 129 | 0.44 |
| Flavobacteriia | 116 | 0.4 |
| Betaproteobacteria | 82 | 0.28 |
| Candidatus Saccharimonia | 31 | 0.11 |
| Clostridia | 24 | 0.08 |
| Mollicutes | 24 | 0.08 |
| Bacteroidia | 14 | 0.05 |

| **Pey10** | | |
| --- | --- | --- |
| **Taxa** | **Count** | **%** |
| Bacilli | 23825 | 96.77 |
| Gammaproteobacteria | 332 | 1.35 |
| Alphaproteobacteria | 111 | 0.45 |
| Actinomycetia | 88 | 0.36 |
| Betaproteobacteria | 82 | 0.33 |
| Flavobacteriia | 58 | 0.24 |
| Candidatus Saccharimonia | 32 | 0.13 |
| Clostridia | 22 | 0.09 |
| Mollicutes | 22 | 0.09 |
| Thermodesulfobacteria <class> | 7 | 0.03 |

| **Pey11** | | |
| --- | --- | --- |
| **Taxa** | **Count** | **%** |
| Bacilli | 25622 | 98.29 |
| Actinomycetia | 180 | 0.69 |
| Alphaproteobacteria | 91 | 0.35 |
| Gammaproteobacteria | 42 | 0.16 |
| Betaproteobacteria | 39 | 0.15 |
| Mollicutes | 38 | 0.15 |
| Clostridia | 24 | 0.09 |
| Thermodesulfobacteria <class> | 6 | 0.02 |
| Erysipelotrichia | 5 | 0.02 |
| Fusobacteriia | 4 | 0.02 |

| **Pey12** | | |
| --- | --- | --- |
| **Taxa** | **Count** | **%** |
| Bacilli | 29459 | 93.73 |
| Gammaproteobacteria | 1567 | 4.99 |
| Alphaproteobacteria | 135 | 0.43 |
| Actinomycetia | 92 | 0.29 |
| Betaproteobacteria | 37 | 0.12 |
| Mollicutes | 28 | 0.09 |
| Clostridia | 25 | 0.08 |
| Planctomycetia | 15 | 0.05 |
| Negativicutes | 9 | 0.03 |
| Thermodesulfobacteria <class> | 8 | 0.03 |

| **Pey13** | | |
| --- | --- | --- |
| **Taxa** | **Count** | **%** |
| Bacilli | 34982 | 90.06 |
| Gammaproteobacteria | 3178 | 8.18 |
| Alphaproteobacteria | 231 | 0.59 |
| Actinomycetia | 185 | 0.48 |
| Betaproteobacteria | 72 | 0.19 |
| Clostridia | 60 | 0.15 |
| Mollicutes | 30 | 0.08 |
| Thermodesulfobacteria <class> | 14 | 0.04 |
| Negativicutes | 11 | 0.03 |
| Bacteroidia | 9 | 0.02 |

| **Pey14** | | |
| --- | --- | --- |
| **Taxa** | **Count** | **%** |
| Bacilli | 11022 | 92.86 |
| Gammaproteobacteria | 281 | 2.37 |
| Betaproteobacteria | 193 | 1.63 |
| Alphaproteobacteria | 184 | 1.55 |
| Actinomycetia | 92 | 0.78 |
| Planctomycetia | 20 | 0.17 |
| Deltaproteobacteria | 19 | 0.16 |
| Flavobacteriia | 13 | 0.11 |
| Clostridia | 12 | 0.1 |
| Mollicutes | 9 | 0.08 |

| **Pey15** | | |
| --- | --- | --- |
| **Taxa** | **Count** | **%** |
| Bacilli | 29103 | 91.25 |
| Gammaproteobacteria | 2060 | 6.46 |
| Actinomycetia | 258 | 0.81 |
| Alphaproteobacteria | 135 | 0.42 |
| Flavobacteriia | 100 | 0.31 |
| Betaproteobacteria | 67 | 0.21 |
| Clostridia | 37 | 0.12 |
| Sphingobacteriia | 29 | 0.09 |
| Mollicutes | 28 | 0.09 |
| Negativicutes | 13 | 0.04 |

| **Pey16** | | |
| --- | --- | --- |
| **Taxa** | **Count** | **%** |
| Bacilli | 15694 | 83.75 |
| Gammaproteobacteria | 2285 | 12.19 |
| Alphaproteobacteria | 300 | 1.6 |
| Actinomycetia | 153 | 0.82 |
| Flavobacteriia | 96 | 0.51 |
| Clostridia | 64 | 0.34 |
| Betaproteobacteria | 59 | 0.31 |
| Mollicutes | 12 | 0.06 |
| Bacteroidia | 11 | 0.06 |
| Spirochaetia | 7 | 0.04 |

| **Pey17** | | |
| --- | --- | --- |
| **Taxa** | **Count** | **%** |
| Bacilli | 40362 | 97.94 |
| Alphaproteobacteria | 243 | 0.59 |
| Actinomycetia | 215 | 0.52 |
| Gammaproteobacteria | 155 | 0.38 |
| Betaproteobacteria | 50 | 0.12 |
| Mollicutes | 46 | 0.11 |
| Clostridia | 43 | 0.1 |
| Spirochaetia | 16 | 0.04 |
| Erysipelotrichia | 9 | 0.02 |
| Bacteroidia | 7 | 0.02 |

| **Pey18** | | |
| --- | --- | --- |
| **Taxa** | **Count** | **%** |
| Bacilli | 164928 | 98.78 |
| Gammaproteobacteria | 991 | 0.59 |
| Actinomycetia | 214 | 0.13 |
| Alphaproteobacteria | 209 | 0.13 |
| Clostridia | 151 | 0.09 |
| Mollicutes | 128 | 0.08 |
| Betaproteobacteria | 79 | 0.05 |
| Flavobacteriia | 45 | 0.03 |
| Erysipelotrichia | 26 | 0.02 |
| Negativicutes | 19 | 0.01 |

| **Pey19** | | |
| --- | --- | --- |
| **Taxa** | **Count** | **%** |
| Bacilli | 48354 | 93.5 |
| Gammaproteobacteria | 1705 | 3.3 |
| Actinomycetia | 896 | 1.73 |
| Mollicutes | 214 | 0.41 |
| Alphaproteobacteria | 171 | 0.33 |
| Erysipelotrichia | 110 | 0.21 |
| Clostridia | 97 | 0.19 |
| Betaproteobacteria | 38 | 0.07 |
| Negativicutes | 17 | 0.03 |
| Planctomycetia | 15 | 0.03 |

| **Pey20** | | |
| --- | --- | --- |
| **Taxa** | **Count** | **%** |
| Bacilli | 24515 | 87.04 |
| Gammaproteobacteria | 2600 | 9.23 |
| Mollicutes | 378 | 1.34 |
| Actinomycetia | 269 | 0.96 |
| Alphaproteobacteria | 196 | 0.7 |
| Betaproteobacteria | 61 | 0.22 |
| Flavobacteriia | 26 | 0.09 |
| Epsilonproteobacteria | 20 | 0.07 |
| Spirochaetia | 13 | 0.05 |
| Clostridia | 13 | 0.05 |

| **Pey21** | | |
| --- | --- | --- |
| **Taxa** | **Count** | **%** |
| Bacilli | 16269 | 92.1 |
| Gammaproteobacteria | 469 | 2.66 |
| Alphaproteobacteria | 218 | 1.23 |
| Actinomycetia | 200 | 1.13 |
| Cytophagia | 161 | 0.91 |
| Erysipelotrichia | 138 | 0.78 |
| Clostridia | 40 | 0.23 |
| Mollicutes | 39 | 0.22 |
| Negativicutes | 26 | 0.15 |
| Betaproteobacteria | 15 | 0.08 |

| **Pey22** | | |
| --- | --- | --- |
| **Taxa** | **Count** | **%** |
| Bacilli | 53521 | 97.31 |
| Gammaproteobacteria | 432 | 0.79 |
| Clostridia | 337 | 0.61 |
| Actinomycetia | 224 | 0.41 |
| Alphaproteobacteria | 212 | 0.39 |
| Mollicutes | 73 | 0.13 |
| Betaproteobacteria | 51 | 0.09 |
| Flavobacteriia | 29 | 0.05 |
| Planctomycetia | 26 | 0.05 |
| Spirochaetia | 17 | 0.03 |

| **Pey23** | | |
| --- | --- | --- |
| **Taxa** | **Count** | **%** |
| Bacilli | 40823 | 94.35 |
| Gammaproteobacteria | 1308 | 3.02 |
| Actinomycetia | 378 | 0.87 |
| Alphaproteobacteria | 269 | 0.62 |
| Betaproteobacteria | 190 | 0.44 |
| Mollicutes | 47 | 0.11 |
| Planctomycetia | 43 | 0.1 |
| Clostridia | 43 | 0.1 |
| Erysipelotrichia | 22 | 0.05 |
| Negativicutes | 19 | 0.04 |

| **Pey24** | | |
| --- | --- | --- |
| **Taxa** | **Count** | **%** |
| Bacilli | 46280 | 96.64 |
| Actinomycetia | 421 | 0.88 |
| Alphaproteobacteria | 335 | 0.7 |
| Mollicutes | 314 | 0.66 |
| Gammaproteobacteria | 240 | 0.5 |
| Clostridia | 51 | 0.11 |
| Tissierellia | 46 | 0.1 |
| Betaproteobacteria | 36 | 0.08 |
| Flavobacteriia | 34 | 0.07 |
| Bacteroidia | 19 | 0.04 |

| **Pey25** | | |
| --- | --- | --- |
| **Taxa** | **Count** | **%** |
| Bacilli | 60554 | 88.16 |
| Gammaproteobacteria | 6808 | 9.91 |
| Actinomycetia | 638 | 0.93 |
| Alphaproteobacteria | 204 | 0.3 |
| Betaproteobacteria | 156 | 0.23 |
| Clostridia | 58 | 0.08 |
| Mollicutes | 50 | 0.07 |
| Flavobacteriia | 25 | 0.04 |
| Erysipelotrichia | 20 | 0.03 |
| Thermodesulfobacteria <class> | 20 | 0.03 |

| **Pey26** | | |
| --- | --- | --- |
| **Taxa** | **Count** | **%** |
| Bacilli | 61897 | 89.45 |
| Gammaproteobacteria | 6346 | 9.17 |
| Actinomycetia | 304 | 0.44 |
| Alphaproteobacteria | 244 | 0.35 |
| Betaproteobacteria | 94 | 0.14 |
| Mollicutes | 72 | 0.1 |
| Clostridia | 53 | 0.08 |
| Flavobacteriia | 51 | 0.07 |
| Erysipelotrichia | 16 | 0.02 |
| Deltaproteobacteria | 14 | 0.02 |

| **Pey27** | | |
| --- | --- | --- |
| **Taxa** | **Count** | **%** |
| Bacilli | 9542 | 84.52 |
| Alphaproteobacteria | 685 | 6.07 |
| Gammaproteobacteria | 654 | 5.79 |
| Actinomycetia | 99 | 0.88 |
| Clostridia | 88 | 0.78 |
| Betaproteobacteria | 82 | 0.73 |
| Flavobacteriia | 34 | 0.3 |
| Cytophagia | 28 | 0.25 |
| Bacteroidia | 14 | 0.12 |
| Mollicutes | 13 | 0.12 |

| **Pey28** | | |
| --- | --- | --- |
| **Taxa** | **Count** | **%** |
| Bacilli | 34493 | 91.95 |
| Gammaproteobacteria | 1211 | 3.23 |
| Alphaproteobacteria | 870 | 2.32 |
| Actinomycetia | 524 | 1.4 |
| Betaproteobacteria | 203 | 0.54 |
| Clostridia | 64 | 0.17 |
| Mollicutes | 55 | 0.15 |
| Planctomycetia | 8 | 0.02 |
| Flavobacteriia | 8 | 0.02 |
| Anaerolineae | 7 | 0.02 |

| **Pey29** | | |
| --- | --- | --- |
| **Taxa** | **Count** | **%** |
| Bacilli | 11328 | 78.35 |
| Mollicutes | 1526 | 10.55 |
| Gammaproteobacteria | 708 | 4.9 |
| Alphaproteobacteria | 565 | 3.91 |
| Actinomycetia | 202 | 1.4 |
| Betaproteobacteria | 61 | 0.42 |
| Deinococci | 14 | 0.1 |
| Clostridia | 10 | 0.07 |
| Fusobacteriia | 7 | 0.05 |
| Planctomycetia | 6 | 0.04 |

| **Pey30** | | |
| --- | --- | --- |
| **Taxa** | **Count** | **%** |
| Bacilli | 28526 | 94.15 |
| Alphaproteobacteria | 737 | 2.43 |
| Gammaproteobacteria | 370 | 1.22 |
| Actinomycetia | 278 | 0.92 |
| Mollicutes | 140 | 0.46 |
| Betaproteobacteria | 99 | 0.33 |
| Clostridia | 41 | 0.14 |
| Erysipelotrichia | 13 | 0.04 |
| Bacteroidia | 11 | 0.04 |
| Flavobacteriia | 8 | 0.03 |

| **Pey31** | | |
| --- | --- | --- |
| **Taxa** | **Count** | **%** |
| Bacilli | 40631 | 94.22 |
| Alphaproteobacteria | 1076 | 2.5 |
| Actinomycetia | 622 | 1.44 |
| Gammaproteobacteria | 491 | 1.14 |
| Betaproteobacteria | 72 | 0.17 |
| Mollicutes | 52 | 0.12 |
| Clostridia | 48 | 0.11 |
| Erysipelotrichia | 30 | 0.07 |
| Spirochaetia | 11 | 0.03 |
| Deltaproteobacteria | 11 | 0.03 |

| **Pey32** | | |
| --- | --- | --- |
| **Taxa** | **Count** | **%** |
| Bacilli | 20734 | 92.54 |
| Alphaproteobacteria | 967 | 4.32 |
| Actinomycetia | 253 | 1.13 |
| Gammaproteobacteria | 187 | 0.83 |
| Betaproteobacteria | 78 | 0.35 |
| Deltaproteobacteria | 48 | 0.21 |
| Clostridia | 29 | 0.13 |
| Mollicutes | 18 | 0.08 |
| Bacteroidia | 16 | 0.07 |
| Spirochaetia | 13 | 0.06 |

| **Pey33** | | |
| --- | --- | --- |
| **Taxa** | **Count** | **%** |
| Bacilli | 19268 | 96.72 |
| Alphaproteobacteria | 338 | 1.7 |
| Gammaproteobacteria | 120 | 0.6 |
| Actinomycetia | 116 | 0.58 |
| Betaproteobacteria | 24 | 0.12 |
| Clostridia | 15 | 0.08 |
| Mollicutes | 9 | 0.05 |
| Bacteroidia | 6 | 0.03 |
| Flavobacteriia | 5 | 0.03 |
| Erysipelotrichia | 4 | 0.02 |

| **Pey34** | | |
| --- | --- | --- |
| **Taxa** | **Count** | **%** |
| Gammaproteobacteria | 3103 | 48.9 |
| Bacilli | 1573 | 24.79 |
| Alphaproteobacteria | 948 | 14.94 |
| Betaproteobacteria | 272 | 4.29 |
| Actinomycetia | 222 | 3.5 |
| Bacteroidia | 63 | 0.99 |
| Clostridia | 41 | 0.65 |
| Spirochaetia | 29 | 0.46 |
| Flavobacteriia | 23 | 0.36 |
| Cytophagia | 12 | 0.19 |

| **Pey35** | | |
| --- | --- | --- |
| **Taxa** | **Count** | **%** |
| Bacilli | 6717 | 90.83 |
| Alphaproteobacteria | 280 | 3.79 |
| Gammaproteobacteria | 171 | 2.31 |
| Actinomycetia | 113 | 1.53 |
| Betaproteobacteria | 41 | 0.55 |
| Clostridia | 14 | 0.19 |
| Erysipelotrichia | 13 | 0.18 |
| Bacteroidia | 7 | 0.09 |
| Mollicutes | 7 | 0.09 |
| Deltaproteobacteria | 6 | 0.08 |

| **Pey36** | | |
| --- | --- | --- |
| **Taxa** | **Count** | **%** |
| Bacilli | 43474 | 93.79 |
| Alphaproteobacteria | 1769 | 3.82 |
| Actinomycetia | 451 | 0.97 |
| Gammaproteobacteria | 412 | 0.89 |
| Betaproteobacteria | 53 | 0.11 |
| Clostridia | 43 | 0.09 |
| Flavobacteriia | 37 | 0.08 |
| Mollicutes | 35 | 0.08 |
| Spirochaetia | 8 | 0.02 |
| Deltaproteobacteria | 8 | 0.02 |

| **Pey37** | | |
| --- | --- | --- |
| **Taxa** | **Count** | **%** |
| Bacilli | 41839 | 79.45 |
| Gammaproteobacteria | 8317 | 15.79 |
| Alphaproteobacteria | 1535 | 2.91 |
| Betaproteobacteria | 442 | 0.84 |
| Actinomycetia | 341 | 0.65 |
| Mollicutes | 27 | 0.05 |
| Clostridia | 25 | 0.05 |
| Spirochaetia | 19 | 0.04 |
| Epsilonproteobacteria | 17 | 0.03 |
| Flavobacteriia | 15 | 0.03 |

**Order**

| **Pey1** | | |
| --- | --- | --- |
| **Taxa** | **Count** | **%** |
| Lactobacillales | 5188 | 83.74 |
| Bacillales | 427 | 6.89 |
| Pseudomonadales | 215 | 3.47 |
| Hyphomicrobiales | 111 | 1.79 |
| Burkholderiales | 49 | 0.79 |
| Eubacteriales | 48 | 0.77 |
| Enterobacterales | 33 | 0.53 |
| Sphingomonadales | 11 | 0.18 |
| Propionibacteriales | 9 | 0.15 |
| Xanthomonadales | 8 | 0.13 |

| **Pey2** | | |
| --- | --- | --- |
| **Taxa** | **Count** | **%** |
| Lactobacillales | 50196 | 93.7 |
| Enterobacterales | 1346 | 2.51 |
| Bacillales | 1063 | 1.98 |
| Hyphomicrobiales | 187 | 0.35 |
| Micrococcales | 166 | 0.31 |
| Eubacteriales | 72 | 0.13 |
| Burkholderiales | 65 | 0.12 |
| Streptomycetales | 45 | 0.08 |
| Mycoplasmatales | 44 | 0.08 |
| Pseudomonadales | 35 | 0.07 |

| **Pey3** | | |
| --- | --- | --- |
| **Taxa** | **Count** | **%** |
| Lactobacillales | 41394 | 96.75 |
| Bacillales | 659 | 1.54 |
| Enterobacterales | 228 | 0.53 |
| Hyphomicrobiales | 104 | 0.24 |
| Mycoplasmatales | 37 | 0.09 |
| Streptomycetales | 35 | 0.08 |
| Pseudomonadales | 30 | 0.07 |
| Neisseriales | 29 | 0.07 |
| Eubacteriales | 28 | 0.07 |
| Propionibacteriales | 25 | 0.06 |

| **Pey4** | | |
| --- | --- | --- |
| **Taxa** | **Count** | **%** |
| Lactobacillales | 27909 | 91.52 |
| Enterobacterales | 866 | 2.84 |
| Bacillales | 792 | 2.6 |
| Hyphomicrobiales | 219 | 0.72 |
| Propionibacteriales | 209 | 0.69 |
| Pseudomonadales | 91 | 0.3 |
| Micrococcales | 52 | 0.17 |
| Streptomycetales | 32 | 0.1 |
| Eubacteriales | 27 | 0.09 |
| Burkholderiales | 26 | 0.09 |

| **Pey5** | | |
| --- | --- | --- |
| **Taxa** | **Count** | **%** |
| Lactobacillales | 26770 | 86.86 |
| Enterobacterales | 1846 | 5.99 |
| Bacillales | 958 | 3.11 |
| Hyphomicrobiales | 261 | 0.85 |
| Pseudomonadales | 202 | 0.66 |
| Propionibacteriales | 138 | 0.45 |
| Streptomycetales | 61 | 0.2 |
| Eubacteriales | 61 | 0.2 |
| Mycoplasmatales | 48 | 0.16 |
| Burkholderiales | 41 | 0.13 |

| **Pey6** | | |
| --- | --- | --- |
| **Taxa** | **Count** | **%** |
| Lactobacillales | 29244 | 91.34 |
| Bacillales | 1618 | 5.05 |
| Hyphomicrobiales | 334 | 1.04 |
| Pseudomonadales | 190 | 0.59 |
| Sphingomonadales | 135 | 0.42 |
| Enterobacterales | 124 | 0.39 |
| Propionibacteriales | 65 | 0.2 |
| Streptomycetales | 32 | 0.1 |
| Mycoplasmatales | 28 | 0.09 |
| Burkholderiales | 26 | 0.08 |

| **Pey8** | | |
| --- | --- | --- |
| **Taxa** | **Count** | **%** |
| Lactobacillales | 19176 | 94.39 |
| Bacillales | 545 | 2.68 |
| Enterobacterales | 178 | 0.88 |
| Hyphomicrobiales | 177 | 0.87 |
| Pseudomonadales | 44 | 0.22 |
| Mycoplasmatales | 22 | 0.11 |
| Aeromonadales | 16 | 0.08 |
| Burkholderiales | 14 | 0.07 |
| Neisseriales | 13 | 0.06 |
| Flavobacteriales | 13 | 0.06 |

| **Pey9** | | |
| --- | --- | --- |
| **Taxa** | **Count** | **%** |
| Lactobacillales | 26299 | 93.97 |
| Bacillales | 806 | 2.88 |
| Hyphomicrobiales | 214 | 0.76 |
| Flavobacteriales | 116 | 0.41 |
| Pseudomonadales | 112 | 0.4 |
| Enterobacterales | 63 | 0.23 |
| Neisseriales | 54 | 0.19 |
| Micrococcales | 37 | 0.13 |
| Candidatus Nanosynbacterales | 31 | 0.11 |
| Burkholderiales | 25 | 0.09 |

| **Pey10** | | |
| --- | --- | --- |
| **Taxa** | **Count** | **%** |
| Lactobacillales | 22707 | 95.04 |
| Bacillales | 462 | 1.93 |
| Pseudomonadales | 178 | 0.74 |
| Enterobacterales | 115 | 0.48 |
| Hyphomicrobiales | 81 | 0.34 |
| Flavobacteriales | 58 | 0.24 |
| Neisseriales | 36 | 0.15 |
| Burkholderiales | 32 | 0.13 |
| Candidatus Nanosynbacterales | 32 | 0.13 |
| Micrococcales | 29 | 0.12 |

| **Pey11** | | |
| --- | --- | --- |
| **Taxa** | **Count** | **%** |
| Lactobacillales | 24474 | 96.81 |
| Bacillales | 418 | 1.65 |
| Hyphomicrobiales | 88 | 0.35 |
| Micrococcales | 67 | 0.27 |
| Mycoplasmatales | 31 | 0.12 |
| Neisseriales | 30 | 0.12 |
| Propionibacteriales | 26 | 0.1 |
| Eubacteriales | 24 | 0.09 |
| Enterobacterales | 19 | 0.08 |
| Pseudomonadales | 14 | 0.06 |

| **Pey12** | | |
| --- | --- | --- |
| **Taxa** | **Count** | **%** |
| Lactobacillales | 27782 | 92.91 |
| Enterobacterales | 1129 | 3.78 |
| Bacillales | 431 | 1.44 |
| Pseudomonadales | 110 | 0.37 |
| Hyphomicrobiales | 108 | 0.36 |
| Aeromonadales | 53 | 0.18 |
| Burkholderiales | 28 | 0.09 |
| Propionibacteriales | 26 | 0.09 |
| Mycoplasmatales | 25 | 0.08 |
| Eubacteriales | 21 | 0.07 |

| **Pey13** | | |
| --- | --- | --- |
| **Taxa** | **Count** | **%** |
| Lactobacillales | 33117 | 89.66 |
| Enterobacterales | 1885 | 5.1 |
| Pseudomonadales | 661 | 1.79 |
| Bacillales | 481 | 1.3 |
| Hyphomicrobiales | 213 | 0.58 |
| Aeromonadales | 107 | 0.29 |
| Eubacteriales | 55 | 0.15 |
| Propionibacteriales | 48 | 0.13 |
| Burkholderiales | 42 | 0.11 |
| Streptomycetales | 19 | 0.05 |

| **Pey14** | | |
| --- | --- | --- |
| **Taxa** | **Count** | **%** |
| Lactobacillales | 10622 | 91.7 |
| Burkholderiales | 169 | 1.46 |
| Bacillales | 157 | 1.36 |
| Hyphomicrobiales | 152 | 1.31 |
| Pasteurellales | 127 | 1.1 |
| Pseudomonadales | 62 | 0.54 |
| Enterobacterales | 47 | 0.41 |
| Streptomycetales | 28 | 0.24 |
| Micrococcales | 23 | 0.2 |
| Gemmatales | 20 | 0.17 |

| **Pey15** | | |
| --- | --- | --- |
| **Taxa** | **Count** | **%** |
| Lactobacillales | 26419 | 88.34 |
| Enterobacterales | 1568 | 5.24 |
| Bacillales | 1115 | 3.73 |
| Pseudomonadales | 150 | 0.5 |
| Micrococcales | 101 | 0.34 |
| Flavobacteriales | 100 | 0.33 |
| Hyphomicrobiales | 77 | 0.26 |
| Eubacteriales | 35 | 0.12 |
| Caulobacterales | 33 | 0.11 |
| Burkholderiales | 33 | 0.11 |

| **Pey16** | | |
| --- | --- | --- |
| **Taxa** | **Count** | **%** |
| Lactobacillales | 15056 | 84.3 |
| Aeromonadales | 829 | 4.64 |
| Enterobacterales | 771 | 4.32 |
| Bacillales | 278 | 1.56 |
| Hyphomicrobiales | 269 | 1.51 |
| Pseudomonadales | 131 | 0.73 |
| Flavobacteriales | 96 | 0.54 |
| Eubacteriales | 61 | 0.34 |
| Propionibacteriales | 57 | 0.32 |
| Alteromonadales | 35 | 0.2 |

| **Pey17** | | |
| --- | --- | --- |
| **Taxa** | **Count** | **%** |
| Lactobacillales | 39064 | 96.86 |
| Bacillales | 451 | 1.12 |
| Hyphomicrobiales | 227 | 0.56 |
| Streptomycetales | 137 | 0.34 |
| Enterobacterales | 85 | 0.21 |
| Eubacteriales | 42 | 0.1 |
| Mycoplasmatales | 39 | 0.1 |
| Pseudomonadales | 31 | 0.08 |
| Neisseriales | 25 | 0.06 |
| Propionibacteriales | 23 | 0.06 |

| **Pey18** | | |
| --- | --- | --- |
| **Taxa** | **Count** | **%** |
| Lactobacillales | 157286 | 97.58 |
| Bacillales | 2109 | 1.31 |
| Pseudomonadales | 305 | 0.19 |
| Enterobacterales | 303 | 0.19 |
| Hyphomicrobiales | 184 | 0.11 |
| Oceanospirillales | 148 | 0.09 |
| Eubacteriales | 128 | 0.08 |
| Mycoplasmatales | 106 | 0.07 |
| Micrococcales | 47 | 0.03 |
| Flavobacteriales | 45 | 0.03 |

| **Pey19** | | |
| --- | --- | --- |
| **Taxa** | **Count** | **%** |
| Lactobacillales | 41606 | 83.66 |
| Bacillales | 5475 | 11.01 |
| Pseudomonadales | 515 | 1.04 |
| Micrococcales | 503 | 1.01 |
| Oceanospirillales | 344 | 0.69 |
| Enterobacterales | 305 | 0.61 |
| Mycoplasmatales | 201 | 0.4 |
| Hyphomicrobiales | 150 | 0.3 |
| Erysipelotrichales | 110 | 0.22 |
| Eubacteriales | 96 | 0.19 |

| **Pey20** | | |
| --- | --- | --- |
| **Taxa** | **Count** | **%** |
| Lactobacillales | 23196 | 86.61 |
| Enterobacterales | 1227 | 4.58 |
| Bacillales | 523 | 1.95 |
| Pseudomonadales | 447 | 1.67 |
| Mycoplasmatales | 376 | 1.4 |
| Aeromonadales | 289 | 1.08 |
| Hyphomicrobiales | 170 | 0.63 |
| Streptomycetales | 78 | 0.29 |
| Actinomycetales | 52 | 0.19 |
| Alteromonadales | 43 | 0.16 |

| **Pey21** | | |
| --- | --- | --- |
| **Taxa** | **Count** | **%** |
| Lactobacillales | 11176 | 66.08 |
| Bacillales | 4515 | 26.7 |
| Hyphomicrobiales | 196 | 1.16 |
| Cytophagales | 161 | 0.95 |
| Enterobacterales | 159 | 0.94 |
| Pseudomonadales | 151 | 0.89 |
| Erysipelotrichales | 138 | 0.82 |
| Micrococcales | 42 | 0.25 |
| Eubacteriales | 39 | 0.23 |
| Mycoplasmatales | 33 | 0.2 |

| **Pey22** | | |
| --- | --- | --- |
| **Taxa** | **Count** | **%** |
| Lactobacillales | 51705 | 96.4 |
| Bacillales | 546 | 1.02 |
| Eubacteriales | 332 | 0.62 |
| Enterobacterales | 265 | 0.49 |
| Hyphomicrobiales | 195 | 0.36 |
| Pseudomonadales | 86 | 0.16 |
| Propionibacteriales | 70 | 0.13 |
| Mycoplasmatales | 64 | 0.12 |
| Micrococcales | 45 | 0.08 |
| Streptomycetales | 43 | 0.08 |

| **Pey23** | | |
| --- | --- | --- |
| **Taxa** | **Count** | **%** |
| Lactobacillales | 38332 | 91.24 |
| Bacillales | 1541 | 3.67 |
| Enterobacterales | 961 | 2.29 |
| Hyphomicrobiales | 233 | 0.55 |
| Burkholderiales | 181 | 0.43 |
| Propionibacteriales | 125 | 0.3 |
| Micrococcales | 111 | 0.26 |
| Pseudomonadales | 55 | 0.13 |
| Pirellulales | 42 | 0.1 |
| Eubacteriales | 42 | 0.1 |

| **Pey24** | | |
| --- | --- | --- |
| **Taxa** | **Count** | **%** |
| Lactobacillales | 44074 | 94.59 |
| Bacillales | 1023 | 2.2 |
| Hyphomicrobiales | 307 | 0.66 |
| Mycoplasmatales | 303 | 0.65 |
| Propionibacteriales | 155 | 0.33 |
| Micrococcales | 125 | 0.27 |
| Enterobacterales | 83 | 0.18 |
| Eubacteriales | 48 | 0.1 |
| Tissierellales | 45 | 0.1 |
| Aeromonadales | 41 | 0.09 |

| **Pey25** | | |
| --- | --- | --- |
| **Taxa** | **Count** | **%** |
| Lactobacillales | 57713 | 86.55 |
| Pseudomonadales | 3322 | 4.98 |
| Enterobacterales | 2645 | 3.97 |
| Bacillales | 1573 | 2.36 |
| Micrococcales | 210 | 0.31 |
| Hyphomicrobiales | 172 | 0.26 |
| Neisseriales | 104 | 0.16 |
| Propionibacteriales | 99 | 0.15 |
| Aeromonadales | 86 | 0.13 |
| Streptomycetales | 81 | 0.12 |

| **Pey26** | | |
| --- | --- | --- |
| **Taxa** | **Count** | **%** |
| Lactobacillales | 58294 | 88.06 |
| Pseudomonadales | 2871 | 4.34 |
| Enterobacterales | 2615 | 3.95 |
| Bacillales | 1440 | 2.18 |
| Hyphomicrobiales | 209 | 0.32 |
| Mycoplasmatales | 61 | 0.09 |
| Micrococcales | 58 | 0.09 |
| Propionibacteriales | 57 | 0.09 |
| Corynebacteriales | 51 | 0.08 |
| Flavobacteriales | 51 | 0.08 |

| **Pey27** | | |
| --- | --- | --- |
| **Taxa** | **Count** | **%** |
| Lactobacillales | 8936 | 82.43 |
| Hyphomicrobiales | 624 | 5.76 |
| Enterobacterales | 317 | 2.92 |
| Bacillales | 266 | 2.45 |
| Pseudomonadales | 214 | 1.97 |
| Eubacteriales | 88 | 0.81 |
| Burkholderiales | 61 | 0.56 |
| Sphingomonadales | 46 | 0.42 |
| Flavobacteriales | 34 | 0.31 |
| Micrococcales | 33 | 0.3 |

| **Pey28** | | |
| --- | --- | --- |
| **Taxa** | **Count** | **%** |
| Lactobacillales | 32662 | 90.27 |
| Hyphomicrobiales | 847 | 2.34 |
| Bacillales | 734 | 2.03 |
| Pseudomonadales | 482 | 1.33 |
| Enterobacterales | 372 | 1.03 |
| Burkholderiales | 177 | 0.49 |
| Micromonosporales | 159 | 0.44 |
| Micrococcales | 147 | 0.41 |
| Pasteurellales | 108 | 0.3 |
| Eubacteriales | 61 | 0.17 |

| **Pey29** | | |
| --- | --- | --- |
| **Taxa** | **Count** | **%** |
| Lactobacillales | 10456 | 75.66 |
| Mycoplasmatales | 1525 | 11.03 |
| Hyphomicrobiales | 539 | 3.9 |
| Enterobacterales | 396 | 2.87 |
| Bacillales | 359 | 2.6 |
| Pseudomonadales | 162 | 1.17 |
| Micrococcales | 49 | 0.35 |
| Burkholderiales | 48 | 0.35 |
| Streptomycetales | 47 | 0.34 |
| Frankiales | 32 | 0.23 |

| **Pey30** | | |
| --- | --- | --- |
| **Taxa** | **Count** | **%** |
| Lactobacillales | 27401 | 92.83 |
| Hyphomicrobiales | 711 | 2.41 |
| Bacillales | 437 | 1.48 |
| Pseudomonadales | 183 | 0.62 |
| Mycoplasmatales | 140 | 0.47 |
| Micrococcales | 106 | 0.36 |
| Neisseriales | 55 | 0.19 |
| Alteromonadales | 48 | 0.16 |
| Oceanospirillales | 43 | 0.15 |
| Propionibacteriales | 41 | 0.14 |

| **Pey31** | | |
| --- | --- | --- |
| **Taxa** | **Count** | **%** |
| Lactobacillales | 34399 | 82.83 |
| Bacillales | 4788 | 11.53 |
| Hyphomicrobiales | 1042 | 2.51 |
| Enterobacterales | 241 | 0.58 |
| Streptomycetales | 231 | 0.56 |
| Micrococcales | 167 | 0.4 |
| Pseudomonadales | 103 | 0.25 |
| Nostocales | 57 | 0.14 |
| Burkholderiales | 47 | 0.11 |
| Eubacteriales | 46 | 0.11 |

| **Pey32** | | |
| --- | --- | --- |
| **Taxa** | **Count** | **%** |
| Lactobacillales | 19872 | 90.97 |
| Hyphomicrobiales | 934 | 4.28 |
| Bacillales | 352 | 1.61 |
| Enterobacterales | 90 | 0.41 |
| Propionibacteriales | 89 | 0.41 |
| Burkholderiales | 71 | 0.33 |
| Pseudomonadales | 58 | 0.27 |
| Streptomycetales | 47 | 0.22 |
| Desulfobacterales | 43 | 0.2 |
| Micrococcales | 37 | 0.17 |

| **Pey33** | | |
| --- | --- | --- |
| **Taxa** | **Count** | **%** |
| Lactobacillales | 18434 | 95.54 |
| Hyphomicrobiales | 323 | 1.67 |
| Bacillales | 223 | 1.16 |
| Enterobacterales | 80 | 0.41 |
| Micrococcales | 46 | 0.24 |
| Pseudomonadales | 30 | 0.16 |
| Propionibacteriales | 25 | 0.13 |
| Eubacteriales | 15 | 0.08 |
| Neisseriales | 12 | 0.06 |
| Corynebacteriales | 11 | 0.06 |

| **Pey34** | | |
| --- | --- | --- |
| **Taxa** | **Count** | **%** |
| Enterobacterales | 2375 | 40.47 |
| Lactobacillales | 1447 | 24.65 |
| Hyphomicrobiales | 897 | 15.28 |
| Burkholderiales | 207 | 3.53 |
| Pseudomonadales | 161 | 2.74 |
| Bacillales | 94 | 1.6 |
| Streptomycetales | 72 | 1.23 |
| Bacteroidales | 63 | 1.07 |
| Propionibacteriales | 49 | 0.83 |
| Rhodocyclales | 42 | 0.72 |

| **Pey35** | | |
| --- | --- | --- |
| **Taxa** | **Count** | **%** |
| Lactobacillales | 6125 | 86.63 |
| Bacillales | 275 | 3.89 |
| Hyphomicrobiales | 256 | 3.62 |
| Pseudomonadales | 114 | 1.61 |
| Streptomycetales | 59 | 0.83 |
| Burkholderiales | 38 | 0.54 |
| Enterobacterales | 29 | 0.41 |
| Micrococcales | 16 | 0.23 |
| Propionibacteriales | 16 | 0.23 |
| Eubacteriales | 14 | 0.2 |

| **Pey36** | | |
| --- | --- | --- |
| **Taxa** | **Count** | **%** |
| Lactobacillales | 40997 | 91.68 |
| Hyphomicrobiales | 1746 | 3.9 |
| Bacillales | 997 | 2.23 |
| Micrococcales | 181 | 0.4 |
| Enterobacterales | 167 | 0.37 |
| Pseudomonadales | 147 | 0.33 |
| Propionibacteriales | 60 | 0.13 |
| Streptomycetales | 56 | 0.13 |
| Eubacteriales | 40 | 0.09 |
| Flavobacteriales | 37 | 0.08 |

| **Pey37** | | |
| --- | --- | --- |
| **Taxa** | **Count** | **%** |
| Lactobacillales | 39942 | 81.45 |
| Enterobacterales | 3265 | 6.66 |
| Aeromonadales | 1931 | 3.94 |
| Hyphomicrobiales | 1507 | 3.07 |
| Bacillales | 661 | 1.35 |
| Pseudomonadales | 443 | 0.9 |
| Burkholderiales | 389 | 0.79 |
| Alteromonadales | 127 | 0.26 |
| Streptomycetales | 104 | 0.21 |
| Oceanospirillales | 104 | 0.21 |

**Family**

| **Pey1** | | |
| --- | --- | --- |
| **Taxa** | **Count** | **%** |
| Lactobacillaceae | 2535 | 42.87 |
| Streptococcaceae | 2414 | 40.83 |
| Staphylococcaceae | 256 | 4.33 |
| Moraxellaceae | 195 | 3.3 |
| Bradyrhizobiaceae | 102 | 1.73 |
| Enterococcaceae | 88 | 1.49 |
| Bacillaceae | 53 | 0.9 |
| Enterobacteriaceae | 22 | 0.37 |
| Comamonadaceae | 22 | 0.37 |
| Lachnospiraceae | 21 | 0.36 |

| **Pey2** | | |
| --- | --- | --- |
| **Taxa** | **Count** | **%** |
| Streptococcaceae | 29032 | 57.14 |
| Lactobacillaceae | 18864 | 37.13 |
| Enterobacteriaceae | 731 | 1.44 |
| Staphylococcaceae | 454 | 0.89 |
| Bacillaceae | 347 | 0.68 |
| Enterococcaceae | 306 | 0.6 |
| Bradyrhizobiaceae | 170 | 0.33 |
| Micrococcaceae | 89 | 0.18 |
| Planococcaceae | 46 | 0.09 |
| Streptomycetaceae | 45 | 0.09 |

| **Pey3** | | |
| --- | --- | --- |
| **Taxa** | **Count** | **%** |
| Lactobacillaceae | 23770 | 57.55 |
| Streptococcaceae | 16044 | 38.84 |
| Staphylococcaceae | 320 | 0.77 |
| Enterococcaceae | 230 | 0.56 |
| Bacillaceae | 203 | 0.49 |
| Enterobacteriaceae | 139 | 0.34 |
| Bradyrhizobiaceae | 101 | 0.24 |
| Mycoplasmataceae | 37 | 0.09 |
| Streptomycetaceae | 35 | 0.08 |
| Neisseriaceae | 29 | 0.07 |

| **Pey4** | | |
| --- | --- | --- |
| **Taxa** | **Count** | **%** |
| Streptococcaceae | 15074 | 52.02 |
| Lactobacillaceae | 11650 | 40.21 |
| Enterobacteriaceae | 460 | 1.59 |
| Staphylococcaceae | 412 | 1.42 |
| Bradyrhizobiaceae | 196 | 0.68 |
| Bacillaceae | 190 | 0.66 |
| Propionibacteriaceae | 140 | 0.48 |
| Enterococcaceae | 134 | 0.46 |
| Nocardioidaceae | 69 | 0.24 |
| Moraxellaceae | 47 | 0.16 |

| **Pey5** | | |
| --- | --- | --- |
| **Taxa** | **Count** | **%** |
| Streptococcaceae | 17103 | 60.26 |
| Lactobacillaceae | 5314 | 18.72 |
| Enterococcaceae | 2783 | 9.81 |
| Enterobacteriaceae | 965 | 3.4 |
| Bacillaceae | 414 | 1.46 |
| Staphylococcaceae | 333 | 1.17 |
| Bradyrhizobiaceae | 223 | 0.79 |
| Moraxellaceae | 133 | 0.47 |
| Nocardioidaceae | 121 | 0.43 |
| Pseudomonadaceae | 69 | 0.24 |

| **Pey6** | | |
| --- | --- | --- |
| **Taxa** | **Count** | **%** |
| Lactobacillaceae | 17351 | 56.51 |
| Streptococcaceae | 9952 | 32.41 |
| Bacillaceae | 1198 | 3.9 |
| Enterococcaceae | 655 | 2.13 |
| Bradyrhizobiaceae | 312 | 1.02 |
| Staphylococcaceae | 265 | 0.86 |
| Pseudomonadaceae | 164 | 0.53 |
| Carnobacteriaceae | 125 | 0.41 |
| Erythrobacteraceae | 120 | 0.39 |
| Enterobacteriaceae | 52 | 0.17 |

| **Pey8** | | |
| --- | --- | --- |
| **Taxa** | **Count** | **%** |
| Streptococcaceae | 14770 | 75.56 |
| Lactobacillaceae | 3638 | 18.61 |
| Bacillaceae | 357 | 1.83 |
| Staphylococcaceae | 155 | 0.79 |
| Bradyrhizobiaceae | 141 | 0.72 |
| Enterobacteriaceae | 102 | 0.52 |
| Enterococcaceae | 83 | 0.42 |
| Moraxellaceae | 30 | 0.15 |
| Rhizobiaceae | 27 | 0.14 |
| Mycoplasmataceae | 22 | 0.11 |

| **Pey9** | | |
| --- | --- | --- |
| **Taxa** | **Count** | **%** |
| Streptococcaceae | 16331 | 60.79 |
| Lactobacillaceae | 8340 | 31.04 |
| Bacillaceae | 582 | 2.17 |
| Enterococcaceae | 549 | 2.04 |
| Bradyrhizobiaceae | 207 | 0.77 |
| Staphylococcaceae | 135 | 0.5 |
| Moraxellaceae | 100 | 0.37 |
| Weeksellaceae | 99 | 0.37 |
| Enterobacteriaceae | 59 | 0.22 |
| Neisseriaceae | 54 | 0.2 |

| **Pey10** | | |
| --- | --- | --- |
| **Taxa** | **Count** | **%** |
| Streptococcaceae | 13798 | 60.51 |
| Lactobacillaceae | 7481 | 32.81 |
| Enterococcaceae | 436 | 1.91 |
| Bacillaceae | 323 | 1.42 |
| Moraxellaceae | 136 | 0.6 |
| Bradyrhizobiaceae | 70 | 0.31 |
| Enterobacteriaceae | 59 | 0.26 |
| Staphylococcaceae | 56 | 0.25 |
| Weeksellaceae | 50 | 0.22 |
| Pseudomonadaceae | 42 | 0.18 |

| **Pey11** | | |
| --- | --- | --- |
| **Taxa** | **Count** | **%** |
| Lactobacillaceae | 14718 | 60.69 |
| Streptococcaceae | 7560 | 31.17 |
| Enterococcaceae | 1119 | 4.61 |
| Bacillaceae | 203 | 0.84 |
| Carnobacteriaceae | 143 | 0.59 |
| Staphylococcaceae | 113 | 0.47 |
| Bradyrhizobiaceae | 85 | 0.35 |
| Micrococcaceae | 38 | 0.16 |
| Mycoplasmataceae | 31 | 0.13 |
| Neisseriaceae | 30 | 0.12 |

| **Pey12** | | |
| --- | --- | --- |
| **Taxa** | **Count** | **%** |
| Lactobacillaceae | 12914 | 45.62 |
| Streptococcaceae | 7569 | 26.74 |
| Enterococcaceae | 6408 | 22.64 |
| Enterobacteriaceae | 405 | 1.43 |
| Bacillaceae | 211 | 0.75 |
| Staphylococcaceae | 109 | 0.39 |
| Moraxellaceae | 95 | 0.34 |
| Bradyrhizobiaceae | 92 | 0.32 |
| Aeromonadaceae | 53 | 0.19 |
| Propionibacteriaceae | 26 | 0.09 |

| **Pey13** | | |
| --- | --- | --- |
| **Taxa** | **Count** | **%** |
| Lactobacillaceae | 14579 | 42.01 |
| Streptococcaceae | 10067 | 29.01 |
| Enterococcaceae | 7404 | 21.33 |
| Enterobacteriaceae | 686 | 1.98 |
| Moraxellaceae | 633 | 1.82 |
| Bacillaceae | 225 | 0.65 |
| Bradyrhizobiaceae | 205 | 0.59 |
| Staphylococcaceae | 135 | 0.39 |
| Aeromonadaceae | 107 | 0.31 |
| Propionibacteriaceae | 43 | 0.12 |

| **Pey14** | | |
| --- | --- | --- |
| **Taxa** | **Count** | **%** |
| Lactobacillaceae | 5856 | 52.89 |
| Streptococcaceae | 4275 | 38.61 |
| Burkholderiaceae | 136 | 1.23 |
| Bradyrhizobiaceae | 134 | 1.21 |
| Pasteurellaceae | 127 | 1.15 |
| Staphylococcaceae | 70 | 0.63 |
| Bacillaceae | 48 | 0.43 |
| Moraxellaceae | 36 | 0.33 |
| Enterococcaceae | 32 | 0.29 |
| Streptomycetaceae | 28 | 0.25 |

| **Pey15** | | |
| --- | --- | --- |
| **Taxa** | **Count** | **%** |
| Lactobacillaceae | 14602 | 51.71 |
| Streptococcaceae | 8706 | 30.83 |
| Enterococcaceae | 2060 | 7.3 |
| Enterobacteriaceae | 985 | 3.49 |
| Staphylococcaceae | 426 | 1.51 |
| Bacillaceae | 355 | 1.26 |
| Moraxellaceae | 139 | 0.49 |
| Carnobacteriaceae | 136 | 0.48 |
| Micrococcaceae | 97 | 0.34 |
| Weeksellaceae | 92 | 0.33 |

| **Pey16** | | |
| --- | --- | --- |
| **Taxa** | **Count** | **%** |
| Streptococcaceae | 11945 | 70.74 |
| Lactobacillaceae | 2288 | 13.55 |
| Aeromonadaceae | 829 | 4.91 |
| Enterobacteriaceae | 370 | 2.19 |
| Enterococcaceae | 272 | 1.61 |
| Bradyrhizobiaceae | 201 | 1.19 |
| Staphylococcaceae | 122 | 0.72 |
| Moraxellaceae | 93 | 0.55 |
| Bacillaceae | 85 | 0.5 |
| Weeksellaceae | 80 | 0.47 |

| **Pey17** | | |
| --- | --- | --- |
| **Taxa** | **Count** | **%** |
| Lactobacillaceae | 32241 | 82.53 |
| Streptococcaceae | 3997 | 10.23 |
| Enterococcaceae | 1585 | 4.06 |
| Bacillaceae | 245 | 0.63 |
| Bradyrhizobiaceae | 221 | 0.57 |
| Streptomycetaceae | 137 | 0.35 |
| Staphylococcaceae | 107 | 0.27 |
| Carnobacteriaceae | 60 | 0.15 |
| Mycoplasmataceae | 39 | 0.1 |
| Enterobacteriaceae | 31 | 0.08 |

| **Pey18** | | |
| --- | --- | --- |
| **Taxa** | **Count** | **%** |
| Lactobacillaceae | 74149 | 47.37 |
| Streptococcaceae | 72448 | 46.29 |
| Aerococcaceae | 2653 | 1.7 |
| Enterococcaceae | 2155 | 1.38 |
| Carnobacteriaceae | 1752 | 1.12 |
| Bacillaceae | 777 | 0.5 |
| Staphylococcaceae | 722 | 0.46 |
| Moraxellaceae | 206 | 0.13 |
| Enterobacteriaceae | 179 | 0.11 |
| Bradyrhizobiaceae | 178 | 0.11 |

| **Pey19** | | |
| --- | --- | --- |
| **Taxa** | **Count** | **%** |
| Lactobacillaceae | 28853 | 60.84 |
| Streptococcaceae | 10645 | 22.45 |
| Staphylococcaceae | 2612 | 5.51 |
| Bacillaceae | 1293 | 2.73 |
| Enterococcaceae | 1256 | 2.65 |
| Moraxellaceae | 504 | 1.06 |
| Micrococcaceae | 362 | 0.76 |
| Halomonadaceae | 338 | 0.71 |
| Mycoplasmataceae | 201 | 0.42 |
| Enterobacteriaceae | 196 | 0.41 |

| **Pey20** | | |
| --- | --- | --- |
| **Taxa** | **Count** | **%** |
| Streptococcaceae | 17845 | 68.89 |
| Lactobacillaceae | 4914 | 18.97 |
| Enterobacteriaceae | 676 | 2.61 |
| Pseudomonadaceae | 410 | 1.58 |
| Mycoplasmataceae | 376 | 1.45 |
| Staphylococcaceae | 293 | 1.13 |
| Aeromonadaceae | 289 | 1.12 |
| Bradyrhizobiaceae | 157 | 0.61 |
| Enterococcaceae | 137 | 0.53 |
| Bacillaceae | 98 | 0.38 |

| **Pey21** | | |
| --- | --- | --- |
| **Taxa** | **Count** | **%** |
| Enterococcaceae | 4966 | 33.68 |
| Lactobacillaceae | 4797 | 32.54 |
| Staphylococcaceae | 2719 | 18.44 |
| Bacillaceae | 474 | 3.21 |
| Streptococcaceae | 241 | 1.63 |
| Bradyrhizobiaceae | 187 | 1.27 |
| Hymenobacteraceae | 161 | 1.09 |
| Moraxellaceae | 144 | 0.98 |
| Carnobacteriaceae | 140 | 0.95 |
| Erysipelotrichaceae | 135 | 0.92 |

| **Pey22** | | |
| --- | --- | --- |
| **Taxa** | **Count** | **%** |
| Lactobacillaceae | 33700 | 64.69 |
| Streptococcaceae | 16309 | 31.31 |
| Enterococcaceae | 358 | 0.69 |
| Clostridiaceae | 294 | 0.56 |
| Bacillaceae | 242 | 0.46 |
| Bradyrhizobiaceae | 186 | 0.36 |
| Enterobacteriaceae | 136 | 0.26 |
| Staphylococcaceae | 134 | 0.26 |
| Mycoplasmataceae | 64 | 0.12 |
| Moraxellaceae | 63 | 0.12 |

| **Pey23** | | |
| --- | --- | --- |
| **Taxa** | **Count** | **%** |
| Lactobacillaceae | 31958 | 78.57 |
| Streptococcaceae | 5310 | 13.05 |
| Staphylococcaceae | 787 | 1.93 |
| Enterobacteriaceae | 504 | 1.24 |
| Bacillaceae | 408 | 1.0 |
| Aerococcaceae | 250 | 0.61 |
| Bradyrhizobiaceae | 220 | 0.54 |
| Enterococcaceae | 173 | 0.43 |
| Burkholderiaceae | 170 | 0.42 |
| Nocardioidaceae | 104 | 0.26 |

| **Pey24** | | |
| --- | --- | --- |
| **Taxa** | **Count** | **%** |
| Lactobacillaceae | 23749 | 52.57 |
| Streptococcaceae | 18696 | 41.38 |
| Staphylococcaceae | 670 | 1.48 |
| Mycoplasmataceae | 303 | 0.67 |
| Bradyrhizobiaceae | 264 | 0.58 |
| Bacillaceae | 217 | 0.48 |
| Enterococcaceae | 180 | 0.4 |
| Aerococcaceae | 160 | 0.35 |
| Nocardioidaceae | 122 | 0.27 |
| Micrococcaceae | 68 | 0.15 |

| **Pey25** | | |
| --- | --- | --- |
| **Taxa** | **Count** | **%** |
| Lactobacillaceae | 29638 | 47.8 |
| Streptococcaceae | 23594 | 38.05 |
| Moraxellaceae | 3197 | 5.16 |
| Enterobacteriaceae | 1015 | 1.64 |
| Carnobacteriaceae | 885 | 1.43 |
| Staphylococcaceae | 846 | 1.36 |
| Enterococcaceae | 432 | 0.7 |
| Aerococcaceae | 348 | 0.56 |
| Bacillaceae | 345 | 0.56 |
| Micrococcaceae | 173 | 0.28 |

| **Pey26** | | |
| --- | --- | --- |
| **Taxa** | **Count** | **%** |
| Streptococcaceae | 34033 | 54.45 |
| Lactobacillaceae | 22079 | 35.32 |
| Moraxellaceae | 2844 | 4.55 |
| Enterobacteriaceae | 1064 | 1.7 |
| Staphylococcaceae | 532 | 0.85 |
| Enterococcaceae | 382 | 0.61 |
| Bacillaceae | 299 | 0.48 |
| Bradyrhizobiaceae | 184 | 0.29 |
| Mycoplasmataceae | 61 | 0.1 |
| Paenibacillaceae | 51 | 0.08 |

| **Pey27** | | |
| --- | --- | --- |
| **Taxa** | **Count** | **%** |
| Streptococcaceae | 5106 | 49.53 |
| Lactobacillaceae | 3315 | 32.16 |
| Bradyrhizobiaceae | 582 | 5.65 |
| Moraxellaceae | 168 | 1.63 |
| Enterococcaceae | 163 | 1.58 |
| Enterobacteriaceae | 128 | 1.24 |
| Bacillaceae | 114 | 1.11 |
| Carnobacteriaceae | 104 | 1.01 |
| Staphylococcaceae | 89 | 0.86 |
| Pseudomonadaceae | 45 | 0.44 |

| **Pey28** | | |
| --- | --- | --- |
| **Taxa** | **Count** | **%** |
| Lactobacillaceae | 14612 | 42.91 |
| Streptococcaceae | 12550 | 36.86 |
| Enterococcaceae | 3593 | 10.55 |
| Bradyrhizobiaceae | 829 | 2.43 |
| Moraxellaceae | 430 | 1.26 |
| Bacillaceae | 256 | 0.75 |
| Staphylococcaceae | 237 | 0.7 |
| Enterobacteriaceae | 183 | 0.54 |
| Carnobacteriaceae | 170 | 0.5 |
| Burkholderiaceae | 163 | 0.48 |

| **Pey29** | | |
| --- | --- | --- |
| **Taxa** | **Count** | **%** |
| Streptococcaceae | 6668 | 50.37 |
| Lactobacillaceae | 3416 | 25.8 |
| Mycoplasmataceae | 1525 | 11.52 |
| Bradyrhizobiaceae | 528 | 3.99 |
| Enterobacteriaceae | 174 | 1.31 |
| Staphylococcaceae | 142 | 1.07 |
| Moraxellaceae | 142 | 1.07 |
| Bacillaceae | 125 | 0.94 |
| Enterococcaceae | 63 | 0.48 |
| Streptomycetaceae | 47 | 0.36 |

| **Pey30** | | |
| --- | --- | --- |
| **Taxa** | **Count** | **%** |
| Streptococcaceae | 12876 | 45.42 |
| Lactobacillaceae | 12691 | 44.77 |
| Bradyrhizobiaceae | 698 | 2.46 |
| Carnobacteriaceae | 465 | 1.64 |
| Enterococcaceae | 319 | 1.13 |
| Staphylococcaceae | 157 | 0.55 |
| Bacillaceae | 149 | 0.53 |
| Moraxellaceae | 144 | 0.51 |
| Mycoplasmataceae | 140 | 0.49 |
| Micrococcaceae | 60 | 0.21 |

| **Pey31** | | |
| --- | --- | --- |
| **Taxa** | **Count** | **%** |
| Streptococcaceae | 18783 | 48.29 |
| Lactobacillaceae | 13762 | 35.38 |
| Staphylococcaceae | 2834 | 7.29 |
| Bradyrhizobiaceae | 1022 | 2.63 |
| Bacillaceae | 583 | 1.5 |
| Aerococcaceae | 275 | 0.71 |
| Enterococcaceae | 260 | 0.67 |
| Streptomycetaceae | 231 | 0.59 |
| Enterobacteriaceae | 156 | 0.4 |
| Micrococcaceae | 128 | 0.33 |

| **Pey32** | | |
| --- | --- | --- |
| **Taxa** | **Count** | **%** |
| Lactobacillaceae | 12811 | 60.27 |
| Carnobacteriaceae | 4202 | 19.77 |
| Streptococcaceae | 2173 | 10.22 |
| Bradyrhizobiaceae | 912 | 4.29 |
| Bacillaceae | 168 | 0.79 |
| Enterococcaceae | 134 | 0.63 |
| Staphylococcaceae | 106 | 0.5 |
| Propionibacteriaceae | 87 | 0.41 |
| Aerococcaceae | 69 | 0.32 |
| Enterobacteriaceae | 56 | 0.26 |

| **Pey33** | | |
| --- | --- | --- |
| **Taxa** | **Count** | **%** |
| Streptococcaceae | 14275 | 75.97 |
| Lactobacillaceae | 3609 | 19.21 |
| Bradyrhizobiaceae | 318 | 1.69 |
| Staphylococcaceae | 111 | 0.59 |
| Enterococcaceae | 94 | 0.5 |
| Bacillaceae | 65 | 0.35 |
| Enterobacteriaceae | 57 | 0.3 |
| Propionibacteriaceae | 24 | 0.13 |
| Moraxellaceae | 20 | 0.11 |
| Micrococcaceae | 16 | 0.09 |

| **Pey34** | | |
| --- | --- | --- |
| **Taxa** | **Count** | **%** |
| Enterobacteriaceae | 1078 | 23.69 |
| Bradyrhizobiaceae | 803 | 17.65 |
| Streptococcaceae | 744 | 16.35 |
| Lactobacillaceae | 587 | 12.9 |
| Comamonadaceae | 159 | 3.49 |
| Enterococcaceae | 90 | 1.98 |
| Pseudomonadaceae | 82 | 1.8 |
| Moraxellaceae | 79 | 1.74 |
| Streptomycetaceae | 72 | 1.58 |
| Brucellaceae | 65 | 1.43 |

| **Pey35** | | |
| --- | --- | --- |
| **Taxa** | **Count** | **%** |
| Streptococcaceae | 5519 | 79.82 |
| Lactobacillaceae | 276 | 3.99 |
| Bradyrhizobiaceae | 245 | 3.54 |
| Enterococcaceae | 216 | 3.12 |
| Staphylococcaceae | 127 | 1.84 |
| Moraxellaceae | 89 | 1.29 |
| Bacillaceae | 86 | 1.24 |
| Streptomycetaceae | 59 | 0.85 |
| Enterobacteriaceae | 27 | 0.39 |
| Oxalobacteraceae | 27 | 0.39 |

| **Pey36** | | |
| --- | --- | --- |
| **Taxa** | **Count** | **%** |
| Streptococcaceae | 27553 | 64.17 |
| Lactobacillaceae | 11682 | 27.21 |
| Bradyrhizobiaceae | 1720 | 4.01 |
| Staphylococcaceae | 448 | 1.04 |
| Bacillaceae | 260 | 0.61 |
| Enterococcaceae | 208 | 0.48 |
| Enterobacteriaceae | 96 | 0.22 |
| Pseudomonadaceae | 95 | 0.22 |
| Aerococcaceae | 94 | 0.22 |
| Micrococcaceae | 90 | 0.21 |

| **Pey37** | | |
| --- | --- | --- |
| **Taxa** | **Count** | **%** |
| Streptococcaceae | 29264 | 62.72 |
| Lactobacillaceae | 9371 | 20.08 |
| Aeromonadaceae | 1931 | 4.14 |
| Enterobacteriaceae | 1923 | 4.12 |
| Bradyrhizobiaceae | 1466 | 3.14 |
| Burkholderiaceae | 332 | 0.71 |
| Pseudomonadaceae | 293 | 0.63 |
| Staphylococcaceae | 259 | 0.56 |
| Enterococcaceae | 222 | 0.48 |
| Moraxellaceae | 150 | 0.32 |

**Genus**

| **Pey1** | | |
| --- | --- | --- |
| **Taxa** | **Count** | **%** |
| Lactococcus | 1726 | 35.3 |
| Companilactobacillus | 1219 | 24.93 |
| Streptococcus | 543 | 11.11 |
| Staphylococcus | 226 | 4.62 |
| Weissella | 224 | 4.58 |
| Acinetobacter | 190 | 3.89 |
| Bradyrhizobium | 94 | 1.92 |
| Enterococcus | 82 | 1.68 |
| Leuconostoc | 63 | 1.29 |
| Lactobacillus | 57 | 1.17 |

| **Pey2** | | |
| --- | --- | --- |
| **Taxa** | **Count** | **%** |
| Lactococcus | 18287 | 44.47 |
| Streptococcus | 8445 | 20.54 |
| Companilactobacillus | 7789 | 18.94 |
| Leuconostoc | 1271 | 3.09 |
| Weissella | 1144 | 2.78 |
| Staphylococcus | 419 | 1.02 |
| Lactiplantibacillus | 377 | 0.92 |
| Lactobacillus | 305 | 0.74 |
| Enterococcus | 280 | 0.68 |
| Fructilactobacillus | 256 | 0.62 |

| **Pey3** | | |
| --- | --- | --- |
| **Taxa** | **Count** | **%** |
| Lactococcus | 12601 | 48.44 |
| Companilactobacillus | 3785 | 14.55 |
| Lentilactobacillus | 2136 | 8.21 |
| Streptococcus | 1995 | 7.67 |
| Lactiplantibacillus | 923 | 3.55 |
| Leuconostoc | 766 | 2.94 |
| Levilactobacillus | 602 | 2.31 |
| Lactobacillus | 418 | 1.61 |
| Staphylococcus | 285 | 1.1 |
| Weissella | 255 | 0.98 |

| **Pey4** | | |
| --- | --- | --- |
| **Taxa** | **Count** | **%** |
| Lactococcus | 9455 | 40.28 |
| Companilactobacillus | 4921 | 20.96 |
| Streptococcus | 4656 | 19.83 |
| Ligilactobacillus | 895 | 3.81 |
| Leuconostoc | 400 | 1.7 |
| Staphylococcus | 377 | 1.61 |
| Weissella | 269 | 1.15 |
| Lactiplantibacillus | 248 | 1.06 |
| Bradyrhizobium | 195 | 0.83 |
| Lactobacillus | 164 | 0.7 |

| **Pey5** | | |
| --- | --- | --- |
| **Taxa** | **Count** | **%** |
| Lactococcus | 14941 | 64.82 |
| Enterococcus | 2754 | 11.95 |
| Leuconostoc | 1382 | 6.0 |
| Streptococcus | 393 | 1.71 |
| Enterobacter | 387 | 1.68 |
| Bacillus <firmicutes> | 294 | 1.28 |
| Staphylococcus | 291 | 1.26 |
| Lactiplantibacillus | 220 | 0.95 |
| Bradyrhizobium | 205 | 0.89 |
| Acinetobacter | 127 | 0.55 |

| **Pey6** | | |
| --- | --- | --- |
| **Taxa** | **Count** | **%** |
| Lactococcus | 7031 | 32.92 |
| Companilactobacillus | 6036 | 28.26 |
| Streptococcus | 2093 | 9.8 |
| Bacillus <firmicutes> | 1159 | 5.43 |
| Leuconostoc | 903 | 4.23 |
| Lactiplantibacillus | 625 | 2.93 |
| Enterococcus | 326 | 1.53 |
| Tetragenococcus | 321 | 1.5 |
| Bradyrhizobium | 301 | 1.41 |
| Lactobacillus | 215 | 1.01 |

| **Pey8** | | |
| --- | --- | --- |
| **Taxa** | **Count** | **%** |
| Lactococcus | 8763 | 56.91 |
| Streptococcus | 4668 | 30.32 |
| Bacillus <firmicutes> | 345 | 2.24 |
| Lactiplantibacillus | 226 | 1.47 |
| Staphylococcus | 150 | 0.97 |
| Bradyrhizobium | 140 | 0.91 |
| Levilactobacillus | 110 | 0.71 |
| Companilactobacillus | 107 | 0.69 |
| Enterococcus | 74 | 0.48 |
| Lactobacillus | 66 | 0.43 |

| **Pey9** | | |
| --- | --- | --- |
| **Taxa** | **Count** | **%** |
| Streptococcus | 8365 | 39.45 |
| Lactococcus | 6746 | 31.82 |
| Companilactobacillus | 1208 | 5.7 |
| Leuconostoc | 729 | 3.44 |
| Bacillus <firmicutes> | 539 | 2.54 |
| Enterococcus | 481 | 2.27 |
| Levilactobacillus | 314 | 1.48 |
| Lactiplantibacillus | 282 | 1.33 |
| Secundilactobacillus | 251 | 1.18 |
| Latilactobacillus | 250 | 1.18 |

| **Pey10** | | |
| --- | --- | --- |
| **Taxa** | **Count** | **%** |
| Streptococcus | 7228 | 40.37 |
| Lactococcus | 5600 | 31.27 |
| Companilactobacillus | 1183 | 6.61 |
| Leuconostoc | 656 | 3.66 |
| Enterococcus | 389 | 2.17 |
| Bacillus <firmicutes> | 291 | 1.63 |
| Levilactobacillus | 283 | 1.58 |
| Secundilactobacillus | 246 | 1.37 |
| Latilactobacillus | 235 | 1.31 |
| Lactiplantibacillus | 231 | 1.29 |

| **Pey11** | | |
| --- | --- | --- |
| **Taxa** | **Count** | **%** |
| Streptococcus | 5661 | 31.5 |
| Leuconostoc | 4891 | 27.22 |
| Lactococcus | 1522 | 8.47 |
| Lactobacillus | 1466 | 8.16 |
| Enterococcus | 1079 | 6.0 |
| Levilactobacillus | 716 | 3.98 |
| Lacticaseibacillus | 403 | 2.24 |
| Companilactobacillus | 377 | 2.1 |
| Lactiplantibacillus | 313 | 1.74 |
| Bacillus <firmicutes> | 183 | 1.02 |

| **Pey12** | | |
| --- | --- | --- |
| **Taxa** | **Count** | **%** |
| Companilactobacillus | 7011 | 30.01 |
| Tetragenococcus | 6275 | 26.86 |
| Lactococcus | 5701 | 24.4 |
| Streptococcus | 1191 | 5.1 |
| Leuconostoc | 491 | 2.1 |
| Fructilactobacillus | 346 | 1.48 |
| Lactiplantibacillus | 240 | 1.03 |
| Lactobacillus | 229 | 0.98 |
| Bacillus <firmicutes> | 136 | 0.58 |
| Enterococcus | 128 | 0.55 |

| **Pey13** | | |
| --- | --- | --- |
| **Taxa** | **Count** | **%** |
| Companilactobacillus | 7842 | 27.1 |
| Tetragenococcus | 7237 | 25.01 |
| Lactococcus | 7033 | 24.3 |
| Streptococcus | 2191 | 7.57 |
| Acinetobacter | 612 | 2.11 |
| Leuconostoc | 524 | 1.81 |
| Fructilactobacillus | 362 | 1.25 |
| Lactiplantibacillus | 340 | 1.17 |
| Lactobacillus | 243 | 0.84 |
| Bradyrhizobium | 205 | 0.71 |

| **Pey14** | | |
| --- | --- | --- |
| **Taxa** | **Count** | **%** |
| Lactococcus | 2141 | 31.67 |
| Streptococcus | 1768 | 26.15 |
| Companilactobacillus | 701 | 10.37 |
| Levilactobacillus | 388 | 5.74 |
| Lactiplantibacillus | 270 | 3.99 |
| Bradyrhizobium | 127 | 1.88 |
| Haemophilus | 126 | 1.86 |
| Leuconostoc | 117 | 1.73 |
| Polynucleobacter | 116 | 1.72 |
| Lactobacillus | 102 | 1.51 |

| **Pey15** | | |
| --- | --- | --- |
| **Taxa** | **Count** | **%** |
| Lactococcus | 7105 | 32.19 |
| Companilactobacillus | 4868 | 22.05 |
| Lactobacillus | 2981 | 13.5 |
| Enterococcus | 1088 | 4.93 |
| Tetragenococcus | 953 | 4.32 |
| Streptococcus | 804 | 3.64 |
| Leuconostoc | 400 | 1.81 |
| Ligilactobacillus | 395 | 1.79 |
| Staphylococcus | 312 | 1.41 |
| Bacillus <firmicutes> | 277 | 1.25 |

| **Pey16** | | |
| --- | --- | --- |
| **Taxa** | **Count** | **%** |
| Streptococcus | 6534 | 44.09 |
| Lactococcus | 4470 | 30.16 |
| Aeromonas | 828 | 5.59 |
| Weissella | 345 | 2.33 |
| Companilactobacillus | 327 | 2.21 |
| Enterococcus | 257 | 1.73 |
| Latilactobacillus | 218 | 1.47 |
| Bradyrhizobium | 194 | 1.31 |
| Leuconostoc | 145 | 0.98 |
| Staphylococcus | 112 | 0.76 |

| **Pey17** | | |
| --- | --- | --- |
| **Taxa** | **Count** | **%** |
| Lactobacillus | 7528 | 28.89 |
| Streptococcus | 3396 | 13.03 |
| Companilactobacillus | 2368 | 9.09 |
| Levilactobacillus | 2301 | 8.83 |
| Tetragenococcus | 1443 | 5.54 |
| Leuconostoc | 1321 | 5.07 |
| Pediococcus | 1212 | 4.65 |
| Weissella | 1029 | 3.95 |
| Loigolactobacillus | 896 | 3.44 |
| Lacticaseibacillus | 813 | 3.12 |

| **Pey18** | | |
| --- | --- | --- |
| **Taxa** | **Count** | **%** |
| Lactococcus | 45544 | 34.82 |
| Lactobacillus | 31779 | 24.3 |
| Streptococcus | 19428 | 14.85 |
| Companilactobacillus | 9722 | 7.43 |
| Weissella | 3916 | 2.99 |
| Latilactobacillus | 3489 | 2.67 |
| Aerococcus | 2648 | 2.02 |
| Leuconostoc | 2551 | 1.95 |
| Ligilactobacillus | 1366 | 1.04 |
| Enterococcus | 1195 | 0.91 |

| **Pey19** | | |
| --- | --- | --- |
| **Taxa** | **Count** | **%** |
| Lactobacillus | 19178 | 44.64 |
| Streptococcus | 8694 | 20.24 |
| Ligilactobacillus | 2766 | 6.44 |
| Companilactobacillus | 2600 | 6.05 |
| Staphylococcus | 2231 | 5.19 |
| Lactococcus | 1570 | 3.65 |
| Bacillus <firmicutes> | 1172 | 2.73 |
| Tetragenococcus | 1001 | 2.33 |
| Acinetobacter | 394 | 0.92 |
| Kocuria | 258 | 0.6 |

| **Pey20** | | |
| --- | --- | --- |
| **Taxa** | **Count** | **%** |
| Lactococcus | 16147 | 69.76 |
| Companilactobacillus | 2382 | 10.29 |
| Leuconostoc | 484 | 2.09 |
| Pseudomonas | 408 | 1.76 |
| Ligilactobacillus | 390 | 1.68 |
| Streptococcus | 388 | 1.68 |
| Enterobacter | 288 | 1.24 |
| Aeromonas | 288 | 1.24 |
| Mycoplasmopsis | 283 | 1.22 |
| Staphylococcus | 265 | 1.14 |

| **Pey21** | | |
| --- | --- | --- |
| **Taxa** | **Count** | **%** |
| Tetragenococcus | 4907 | 37.85 |
| Ligilactobacillus | 2723 | 21.0 |
| Staphylococcus | 2227 | 17.18 |
| Bacillus <firmicutes> | 388 | 2.99 |
| Companilactobacillus | 322 | 2.48 |
| Bradyrhizobium | 186 | 1.43 |
| Streptococcus | 168 | 1.3 |
| Rufibacter | 161 | 1.24 |
| Weissella | 131 | 1.01 |
| Acinetobacter | 129 | 1.0 |

| **Pey22** | | |
| --- | --- | --- |
| **Taxa** | **Count** | **%** |
| Companilactobacillus | 10187 | 25.25 |
| Lactococcus | 8699 | 21.56 |
| Lactobacillus | 7310 | 18.12 |
| Streptococcus | 6318 | 15.66 |
| Leuconostoc | 2894 | 7.17 |
| Lactiplantibacillus | 626 | 1.55 |
| Levilactobacillus | 551 | 1.37 |
| Lacticaseibacillus | 339 | 0.84 |
| Clostridium | 281 | 0.7 |
| Fructilactobacillus | 272 | 0.67 |

| **Pey23** | | |
| --- | --- | --- |
| **Taxa** | **Count** | **%** |
| Lactobacillus | 16116 | 47.68 |
| Companilactobacillus | 6849 | 20.26 |
| Streptococcus | 4242 | 12.55 |
| Ligilactobacillus | 1484 | 4.39 |
| Lactococcus | 702 | 2.08 |
| Staphylococcus | 627 | 1.86 |
| Bacillus <firmicutes> | 343 | 1.01 |
| Leuconostoc | 320 | 0.95 |
| Enterobacter | 281 | 0.83 |
| Aerococcus | 250 | 0.74 |

| **Pey24** | | |
| --- | --- | --- |
| **Taxa** | **Count** | **%** |
| Streptococcus | 11706 | 31.87 |
| Lactococcus | 5611 | 15.28 |
| Companilactobacillus | 5059 | 13.77 |
| Lactobacillus | 4707 | 12.82 |
| Leuconostoc | 3344 | 9.11 |
| Lacticaseibacillus | 1230 | 3.35 |
| Lentilactobacillus | 717 | 1.95 |
| Staphylococcus | 659 | 1.79 |
| Paucilactobacillus | 293 | 0.8 |
| Lactiplantibacillus | 287 | 0.78 |

| **Pey25** | | |
| --- | --- | --- |
| **Taxa** | **Count** | **%** |
| Streptococcus | 12808 | 27.43 |
| Lactococcus | 9244 | 19.8 |
| Leuconostoc | 4507 | 9.65 |
| Companilactobacillus | 4052 | 8.68 |
| Psychrobacter | 2803 | 6.0 |
| Ligilactobacillus | 1769 | 3.79 |
| Lactiplantibacillus | 1354 | 2.9 |
| Lactobacillus | 1208 | 2.59 |
| Marinilactibacillus | 621 | 1.33 |
| Loigolactobacillus | 585 | 1.25 |

| **Pey26** | | |
| --- | --- | --- |
| **Taxa** | **Count** | **%** |
| Lactococcus | 24735 | 50.27 |
| Lactobacillus | 7580 | 15.41 |
| Streptococcus | 5576 | 11.33 |
| Acinetobacter | 2607 | 5.3 |
| Leuconostoc | 1147 | 2.33 |
| Levilactobacillus | 943 | 1.92 |
| Weissella | 883 | 1.79 |
| Lentilactobacillus | 602 | 1.22 |
| Lactiplantibacillus | 535 | 1.09 |
| Companilactobacillus | 369 | 0.75 |

| **Pey27** | | |
| --- | --- | --- |
| **Taxa** | **Count** | **%** |
| Lactococcus | 3994 | 45.43 |
| Companilactobacillus | 1081 | 12.3 |
| Weissella | 708 | 8.05 |
| Streptococcus | 648 | 7.37 |
| Bradyrhizobium | 557 | 6.34 |
| Lactobacillus | 354 | 4.03 |
| Acinetobacter | 156 | 1.77 |
| Enterococcus | 137 | 1.56 |
| Marinilactibacillus | 84 | 0.96 |
| Staphylococcus | 81 | 0.92 |

| **Pey28** | | |
| --- | --- | --- |
| **Taxa** | **Count** | **%** |
| Companilactobacillus | 7372 | 25.65 |
| Streptococcus | 5891 | 20.5 |
| Lactococcus | 5775 | 20.09 |
| Tetragenococcus | 3417 | 11.89 |
| Weissella | 957 | 3.33 |
| Bradyrhizobium | 824 | 2.87 |
| Lactobacillus | 557 | 1.94 |
| Leuconostoc | 423 | 1.47 |
| Acinetobacter | 231 | 0.8 |
| Pediococcus | 230 | 0.8 |

| **Pey29** | | |
| --- | --- | --- |
| **Taxa** | **Count** | **%** |
| Lactococcus | 4448 | 40.42 |
| Streptococcus | 1567 | 14.24 |
| Lactobacillus | 1270 | 11.54 |
| Mycoplasmopsis | 1205 | 10.95 |
| Bradyrhizobium | 522 | 4.74 |
| Companilactobacillus | 393 | 3.57 |
| Weissella | 147 | 1.34 |
| Pediococcus | 110 | 1.0 |
| Staphylococcus | 77 | 0.7 |
| Lactiplantibacillus | 67 | 0.61 |

| **Pey30** | | |
| --- | --- | --- |
| **Taxa** | **Count** | **%** |
| Streptococcus | 11539 | 52.09 |
| Companilactobacillus | 3523 | 15.91 |
| Weissella | 1317 | 5.95 |
| Lactobacillus | 910 | 4.11 |
| Leuconostoc | 842 | 3.8 |
| Bradyrhizobium | 693 | 3.13 |
| Lactococcus | 526 | 2.37 |
| Marinilactibacillus | 341 | 1.54 |
| Lactiplantibacillus | 330 | 1.49 |
| Enterococcus | 303 | 1.37 |

| **Pey31** | | |
| --- | --- | --- |
| **Taxa** | **Count** | **%** |
| Lactococcus | 12293 | 40.33 |
| Streptococcus | 4635 | 15.21 |
| Companilactobacillus | 2141 | 7.02 |
| Staphylococcus | 2029 | 6.66 |
| Weissella | 1787 | 5.86 |
| Leuconostoc | 1686 | 5.53 |
| Bradyrhizobium | 1017 | 3.34 |
| Ligilactobacillus | 559 | 1.83 |
| Bacillus <firmicutes> | 467 | 1.53 |
| Macrococcus | 364 | 1.19 |

| **Pey32** | | |
| --- | --- | --- |
| **Taxa** | **Count** | **%** |
| Companilactobacillus | 8126 | 49.43 |
| Marinilactibacillus | 2981 | 18.13 |
| Lactococcus | 1077 | 6.55 |
| Streptococcus | 974 | 5.92 |
| Bradyrhizobium | 902 | 5.49 |
| Fructilactobacillus | 471 | 2.86 |
| Leuconostoc | 182 | 1.11 |
| Levilactobacillus | 143 | 0.87 |
| Bacillus <firmicutes> | 133 | 0.81 |
| Lactobacillus | 108 | 0.66 |

| **Pey33** | | |
| --- | --- | --- |
| **Taxa** | **Count** | **%** |
| Lactococcus | 10999 | 69.64 |
| Streptococcus | 2064 | 13.07 |
| Weissella | 545 | 3.45 |
| Levilactobacillus | 426 | 2.7 |
| Companilactobacillus | 395 | 2.5 |
| Bradyrhizobium | 316 | 2.0 |
| Lactiplantibacillus | 107 | 0.68 |
| Staphylococcus | 100 | 0.63 |
| Enterococcus | 92 | 0.58 |
| Lacticaseibacillus | 70 | 0.44 |

| **Pey34** | | |
| --- | --- | --- |
| **Taxa** | **Count** | **%** |
| Bradyrhizobium | 796 | 19.13 |
| Enterobacter | 519 | 12.48 |
| Lactococcus | 460 | 11.06 |
| Ligilactobacillus | 279 | 6.71 |
| Streptococcus | 218 | 5.24 |
| Salmonella | 153 | 3.68 |
| Klebsiella | 130 | 3.13 |
| Escherichia | 113 | 2.72 |
| Companilactobacillus | 89 | 2.14 |
| Enterococcus | 88 | 2.12 |

| **Pey35** | | |
| --- | --- | --- |
| **Taxa** | **Count** | **%** |
| Lactococcus | 4939 | 78.53 |
| Bradyrhizobium | 243 | 3.86 |
| Enterococcus | 211 | 3.36 |
| Streptococcus | 149 | 2.37 |
| Staphylococcus | 111 | 1.76 |
| Acinetobacter | 86 | 1.37 |
| Bacillus <firmicutes> | 53 | 0.84 |
| Streptomyces | 34 | 0.54 |
| Kitasatospora | 25 | 0.4 |
| Pseudomonas | 24 | 0.38 |

| **Pey36** | | |
| --- | --- | --- |
| **Taxa** | **Count** | **%** |
| Lactococcus | 15551 | 45.3 |
| Streptococcus | 9396 | 27.37 |
| Companilactobacillus | 1875 | 5.46 |
| Bradyrhizobium | 1707 | 4.97 |
| Levilactobacillus | 1157 | 3.37 |
| Leuconostoc | 866 | 2.52 |
| Weissella | 426 | 1.24 |
| Lactiplantibacillus | 390 | 1.14 |
| Staphylococcus | 342 | 1.0 |
| Lactobacillus | 287 | 0.84 |

| **Pey37** | | |
| --- | --- | --- |
| **Taxa** | **Count** | **%** |
| Lactococcus | 19020 | 49.48 |
| Streptococcus | 7759 | 20.18 |
| Aeromonas | 1920 | 4.99 |
| Bradyrhizobium | 1451 | 3.77 |
| Pediococcus | 1115 | 2.9 |
| Levilactobacillus | 642 | 1.67 |
| Weissella | 562 | 1.46 |
| Leuconostoc | 538 | 1.4 |
| Loigolactobacillus | 405 | 1.05 |
| Companilactobacillus | 379 | 0.99 |

**Referanslar**

OmicsBox - Bioinformatics made easy. BioBam Bioinformatics (Version 2.0.10). March 3, 2019. [www.](https://www.biobam.com/omicsbox) [biobam.com/omicsbox](https://www.biobam.com/omicsbox).

Wood DE., Lu J. and Langmead B. (2019). Improved metagenomic analysis with Kraken 2. *Genome biology, 20*(1), 257.
